# Synthesis and Characterization of Ternary Manganese–Nickel–Iron Prussian Blue Analogues: Bridging Coordination Chemistry and Solid-State Physics

**DOI:** 10.1021/acsomega.6c01289

**Published:** 2026-05-05

**Authors:** Isabella Concina, Alessio Mezzi, Shujie You

**Affiliations:** † Department of Engineering Sciences and Mathematics, 5185 Luleå University of Technology, 97187 Luleå, Sweden; ‡ CNR, Istituto per lo Studio dei Materiali Nanostrutturati, Strada Provinciale 35d, n. 9, 00010 Montelibretti (RM), Italy

## Abstract

Ternary Prussian blue analogues (*t*-PBAs) containing nickel, manganese, and iron were synthesized via a facile aqueous coprecipitation method to investigate how Mn/Ni ratios influence structural and electrochemical properties. X-ray diffraction, combined with thermal and spectroscopic analyses, showed a systematic dependence of structural parameters on the manganese content, which also impacts the number of coordinated water molecules, suggesting a less defective Fe­(CN)_6_ framework, and weakens the Fe–CN bond. The kinetics and thermodynamics of Ni^2+^ and Mn^2+^ ions in water during nucleation were correlated with ligand-exchange behavior, clarifying their impact on crystalline coherence. The presence of nickel was found to be the key to stabilizing the materials against changes induced by light irradiation and bias solicitation. Indeed, electrochemical measurements demonstrated that Ni-rich analogues exhibit superior stability and capacitance retention, whereas Mn-rich counterparts show an increased capacitive behavior but poorer cycling durability. These findings bridge coordination chemistry and solid-state physics in *t*-PBAs, providing design guidelines for mixed-metal frameworks with tunable properties.

## Graphical abstract

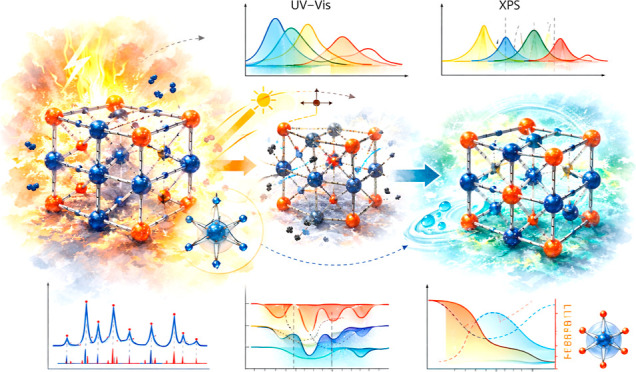

## 1 Introduction

Prussian blue analogues (PBAs) have recently emerged as a promising class of functional materials, with great potential in different applications, spanning from catalysts for clean fuel production to electrochemical energy storage device elements.^1–4^ They are a class of intervalent metal complexes where two transition metal ions (TMIs) are bridged together by a cyanide ligand, whose triple bond is a very efficient shuttle for electron exchange, opening the door up to their potential exploitation in several domains. Their general formula is A_n_M_*y*_[M′(CN)_6_]_*x*_ · H_2_O, where A is an alkali metal and M and M′ are transition metals in different oxidation states.

Despite being known for almost three centuries, their chemistry and physics are still poorly understood. This is even more true when ternary analogues are prepared, where an additional transition metal ion is placed in the structure, which are seldom investigated. These materials are nowadays of particular interest: the insertion of a third transition metal ion can induce significant differences in functionalities, potentially making them flexible platforms in diverse applications.^5–12^

In this study, a series of ternary PBAs, K_*x*_M_3_[Fe­(CN)_6_]_2_ · z H_2_O (where M = Ni_3‑y_Mn_*y*_), were synthesized and characterized: fixing the iron ion as [Fe­(CN)_6_]^3–^, at least nominally, isolates how M-site chemistry and physics govern structure, defect landscape, and behavior upon external solicitations, like light irradiation and bias application.

## 2 Experimental Section

### 2.1 Materials

All of the chemicals were purchased from Sigma-Aldrich and used without any further purification.

### 2.2 Synthesis of Ternary Prussian Blue Analogues

*t*-PBAs were prepared by a coprecipitation method. Stock solutions of K_3_[Fe­(CN)_6_] (0.04 M), Co­(NO_3_)_2_·6 H_2_O (0.08 M), and Ni­(NO_3_)_2_·4 H_2_O (0.08 M) in distilled water were prepared. 50 mL of the K_3_[Fe­(CN)_6_] stock solution was poured into a glass and kept under magnetic stirring (450 rpm). Mn­(NO_3_)_2_·4 H_2_O and Ni­(NO_3_)_2_·4 H_2_O solutions were then dropped in an alternate fashion with a dropping speed of 1 mL/min in such amounts to reach the following Mn/Ni ratio: 0/1, 1/3, 1/1, 3/1, 1/0.

### 2.3 Materials Characterization

#### 2.3.1 X-ray Diffraction (XRD) Analysis

Powder XRD patterns of the samples were recorded on a PANanalytical Empyrean instrument equipped with a PIXcel3D detector and operated at 40 kV and 45 mA using Cu Kα radiation. The obtained powder XRD data were analyzed using High Score Plus software and the Profex Rietveld refinement program.^13^

#### 2.3.2 Scanning Electron Microscopy and Energy-Dispersive X-ray Spectroscopy (EDX)

Energy-dispersive X-ray spectroscopy (EDX) was carried out with a scanning electron microscope (JSM-IT300). AZtech Oxford software was used for the extraction of EDX results from raw data. The appropriate amount of the samples was mounted on a metal plate using Leit-C conductive carbon (Plano GmbH, Wetzlar, Germany) prior to insertion into the microscope.

#### 2.3.3 X-ray Photoelectron Spectroscopy

The surface chemical composition of the samples was investigated by an ESCALAB MkII apparatus, equipped with a standard twin-anode Al/Mg X-ray source and a five-channel Tron detection device. To avoid overlap between the Ni LMM Auger signals and the Co 2p_3/2_ core-level peak signal, a Mg X-ray source (*h*ν = 1253.6 eV) was preferred to an Al X-ray source (*h*ν = 1486.6 eV). All samples were mounted by fixing the powder to a biadhesive carbon disc, taking care to spread the powder to completely cover the carbon disc. All spectra were registered operating at a constant pass energy of 40 eV; the accuracy of the BE value was ±0.1 eV, while the BE scale was calibrated by positioning the contribution of adventitious carbon at 285.0 eV.

All spectra were collected and processed with Avantage v.5.9 software. More details are reported elsewhere.^14^

#### 2.3.4 Fourier Transform Infrared Spectroscopy

Diffuse reflectance Fourier transform–infrared (DRIFT) analysis was performed using a Bruker VERTEX 70v FTIR spectrometer (Germany). Each sample was analyzed in the range of 4000–400 cm^–1^ by averaging 128 scans at a spectral resolution of 2 cm^–1^.

#### 2.3.5 Diffuse Reflectance Spectroscopy

DRS spectra of precursors and synthesized materials were recorded in an Agilent Cary 5000 spectrophotometer, using an integrating sphere.

#### 2.3.6 Electrochemical Measurements

Each *t*-PBA (5 mg) was mixed with mesoporous carbon (2.5 mg) and dispersed in 5 mL of ethanol (containing 0.6% weight PTFE) to obtain a slurry. This latter was sonicated at RT for 90 min. 40 μL of the slurry was drop-cast on a glassy carbon (GC) electrode (3 mm diameter) and let dry in ambient conditions (weight of active material deposited: 3.883 × 10^–2^ mg).

Electrochemical measurements were carried out in a three-electrode configuration, featuring standard calomel as the reference electrode (RE), graphite as the counter electrode (CE), and the PBAs on glassy carbon as the working electrode (WE). K_2_SO_4_ or Na_2_SO_4_ in water (0.5 M) was used as the electrolyte. All of the measurements were carried out in a multipurpose electrochemical station (Solartron Analytical, ModulabXM). Cyclic voltammetry (CV) was recorded by scanning the potential from −0.5 to +1 V vs SCE at different rates (100, 50, 25, and 5 mV/s) for 5 cycles. Material stability under bias solicitation was evaluated by scanning the WE at 100 mV/s for 200 cycles and scanning the potential from −1 to +1 V vs SCE.

## 3 Results and Discussion

### 3.1 Structural, Morphological, and Compositional Analysis

Figure 1a reports the XRD patterns of the prepared materials. The XRD from all Ni-samples can be well indexed using a FCC densely packed, extremely symmetric FCC structure (space group 225, Fm3̅m, the same as the forefather of this class of material, Prussian blue).^15^ With increasing Mn content, the XRD patterns of MnNi–PBAs exhibit a clear, monotonic shift toward lower diffraction angles, accompanied by a slight narrowing of the peak widths. This behavior indicates lattice expansion and increased crystallite size (or reduced lattice strain).

**Figure 1:**
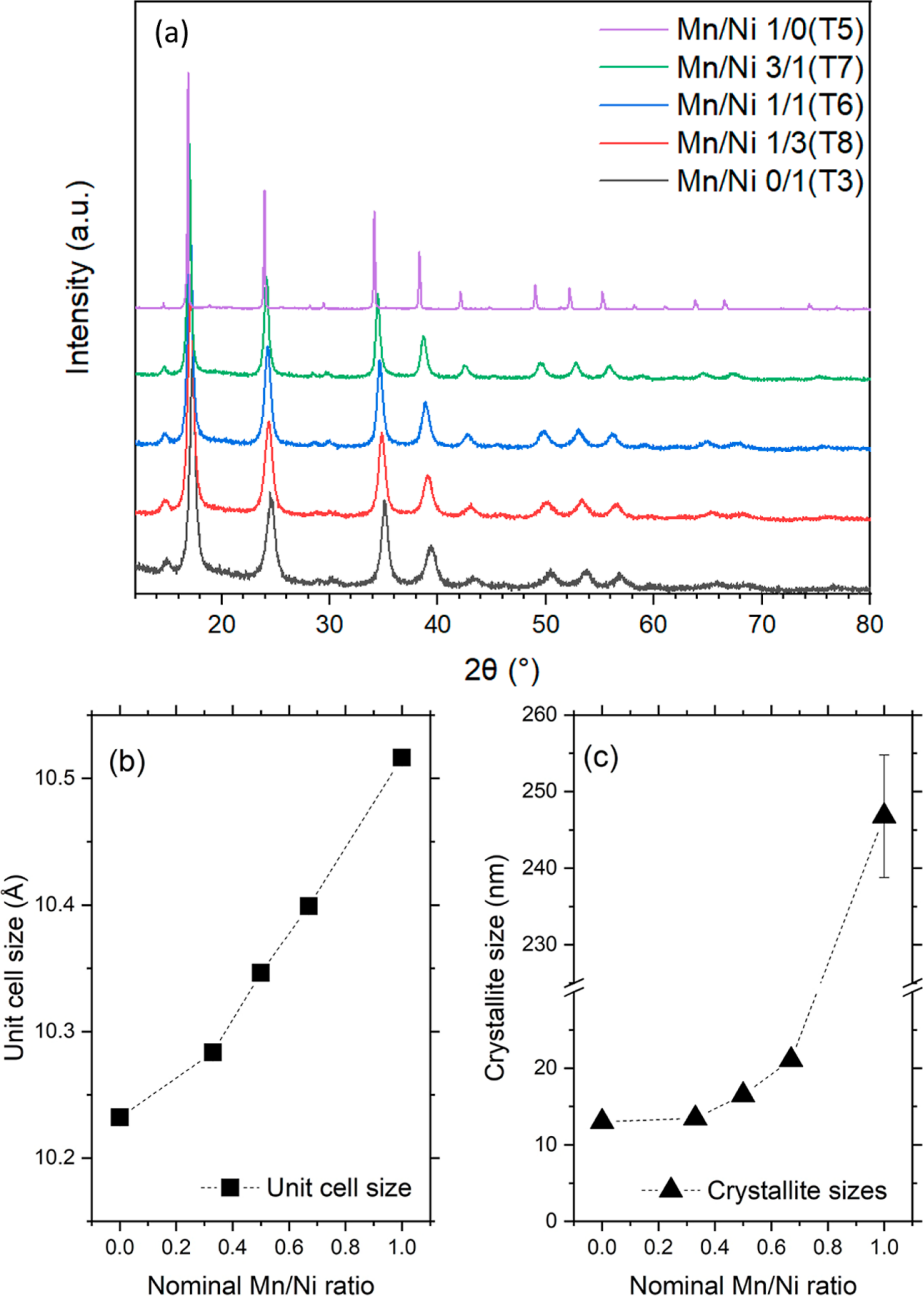
(a) XRD patterns of the PBAs; (b) unit cell size and (c) crystallite sizes as a function of the ratio Mn to Ni. Markers are experimental points, and lines are a guide to the eye.

This trend reaches an extreme at the Y-rich end composition. Meanwhile, several new and broadened peaks emerge at 2θ ≈ 8.41°, 11.90°, 18.86°, 20,68°, 25.40°, 26.80°, as shown in Figures 1a and S1a in the Supporting Information. These reflections can be indexed as the (100), (110), (210), (211), (22,1), and (310) planes of a cubic structure with a lattice parameter of *a* ≈ 10.5163 Å, which are systematically forbidden in an FCC lattice. Their appearance indicates an order–disorder transition accompanied by a loss of the high-symmetry FCC structure and a symmetry reduction to a primitive cubic *Pm*3̅m phase (space group No. 221).

This finding well correlates with the ionic radii tabulated for Ni^2+^ (0.69 Å) and for Mn^2+^ with a coordination number equal to 6 (low spin: 0.81 Å and high spin: 0.97 Å)^16^ so that an increase in the amount of the transition metal ion with a higher ionic radius would result in a stretching of the lattice parameter. Furthermore, considering the electronic configuration of the aquo complexes, being Ni^2+^ a d^8^ and Mn^2+^ a d^5^, water substitution in favor of a high field ligand, such as CN^–^, results in a moderate crystal field stabilization energy for Mn^2+^.

The extension of coherent crystalline domains (Figure 1c and Table 1) was also significantly affected by the insertion of manganese, with a 5-fold increase moving from the binary nickel analogue to the binary manganese composite. Ternary analogues showed extension between the two extreme values, with an increase observable from a Ni/Mn ratio of 1.

**Table 1:** Structural Parameters as Calculated from XRD Analysis

sample label	nominal Mn/Ni ratio	unit cell size (Å)	crystallite size ()
T3	0/1	10.2322 ± 0.0017	13.01 ± 0.12
T8	1/3	10.2834 ± 0.0013	13.46 ± 0.10
T6	1/1	10.3465 ± 0.0011	16.51 ± 0.13
T7	3/1	10.3990 ± 0.0009	21.09 ± 0.18
T5	1/0	10.5163 ± 0.0001 (cubic 225)	246.8 ± 8.0
		10.5163 (cubic 221)	21.22

We analyzed this trend in the light of the inertia toward ligand exchange shown by the aquo complexes of Ni^2+^ and Mn^2+^. Once dissolved in water, the nitrate salts leave room for the formation of the aquo complexes [Ni­(H_2_O)_6_]^2+^ and [Mn­(H_2_O)_6_]^2+^, which then undergo ligand substitution to bind the nitrogen atom of the CN^–^ ligands in the hexacyanoferrate ions available in solution. It is well known from coordination chemistry that the kinetics of ligand exchange in aquo complexes vary significantly in the series of transition metals, with Mn^2+^ being more labile compared to Ni^2+^. Helm and Merbach^17^ have reported the water exchange constants (at 25 °C) for the aquo complexes of Mn^2+^ (2.1 × 10^7^ s^–1^) and Ni^2+^ (1 × 10^4^ s^–1^), clearly showing the lability of Mn^2+^ compared to Ni^2+^. This fact should in principle result in faster nucleation when Mn^2+^ is the dominant species, associated with smaller crystallite sizes (and vice versa, when Ni^2+^ is in higher amounts). This apparent contradiction between the general expectation for kinetic constants can be explained by considering other parameters governing the formation reaction. In the same paper, the authors report the values of the entropy of activation associated with the ligand exchange, which is −22 J mol^–1^ K^–1^ for the nickel hexa–aquo complex and of 12 J mol^–1^ K^–1^, suggesting a dissociative mechanism for ligand substitution in case of nickel and a dissociative interchange mechanism for manganese, with a relative stabilization of the transition state, which supports the formation of more extended crystals.

Morphological analysis through SEM highlighted relevant differences associated with the composition. Moving from the binary analogue containing nickel to that containing manganese (Figure 2), the observed grains showed changes in dimension, together with the formation of cubic microstructures, which is typical for Mn_2_[Fe­(CN)_6_]_3_.

**Figure 2:**
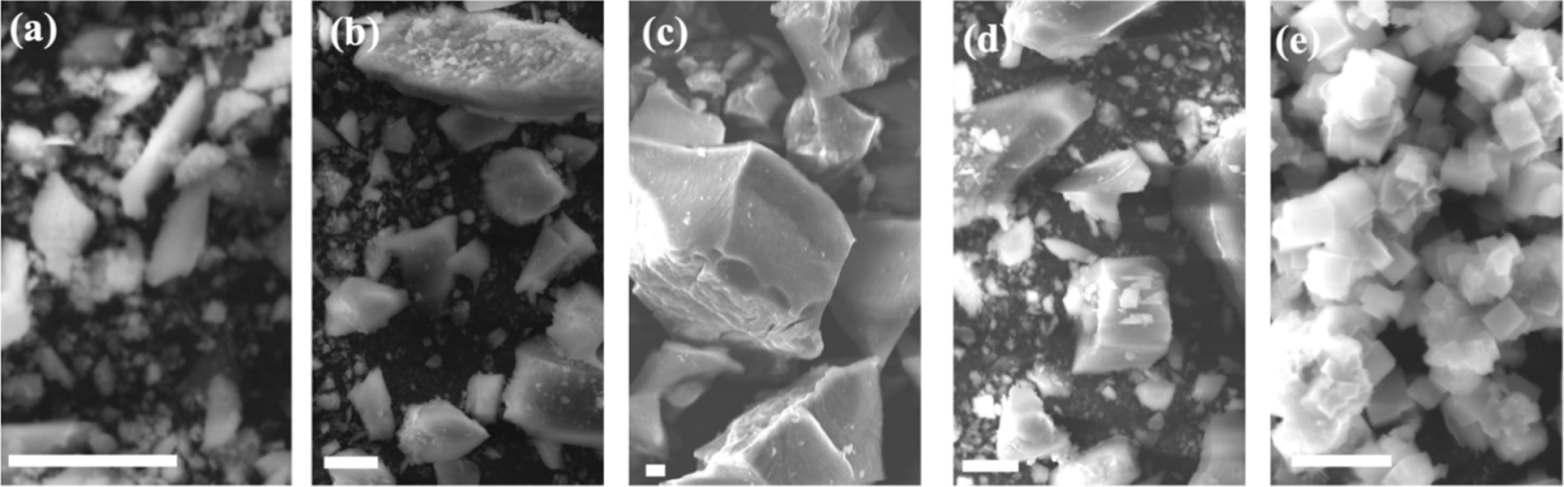
SEM images of the PBA series, from (a) to (e), increasing manganese content. (a) Mn/Ni 0/1; (b) Mn/Ni 1/3; (c) Mn/Ni 1/1; (d) Mn/Ni 3/1; (e) Mn/Ni 1/0. Scale bar: 10 μm.

A relevant characteristic of PBAs is the amount of water present in the structure. Water molecules included in the structure can be of three kinds: adsorbed (or zeolitic) water (present in the structure pores), coordinated water (acting as a ligand to compensate for the vacancies of the Fe­(CN)_6_ units), and hydrogen-bonded water.^18^ Thermal analyses are a powerful tool to discriminate between zeolitic and coordinated water molecules, whose loss occurs at different temperatures. The results pertaining to thermal analyses are shown in Figure 3, and quantification of the water amount is reported in Table 2. The total water content showed a linear decrease with the increase of the amount of manganese, except for the binary nickel analogue, for which the content was slightly lower. Most of these water molecules are adsorbed, while the amount of coordinated water (in weight %) is below 7%.

**Figure 3:**
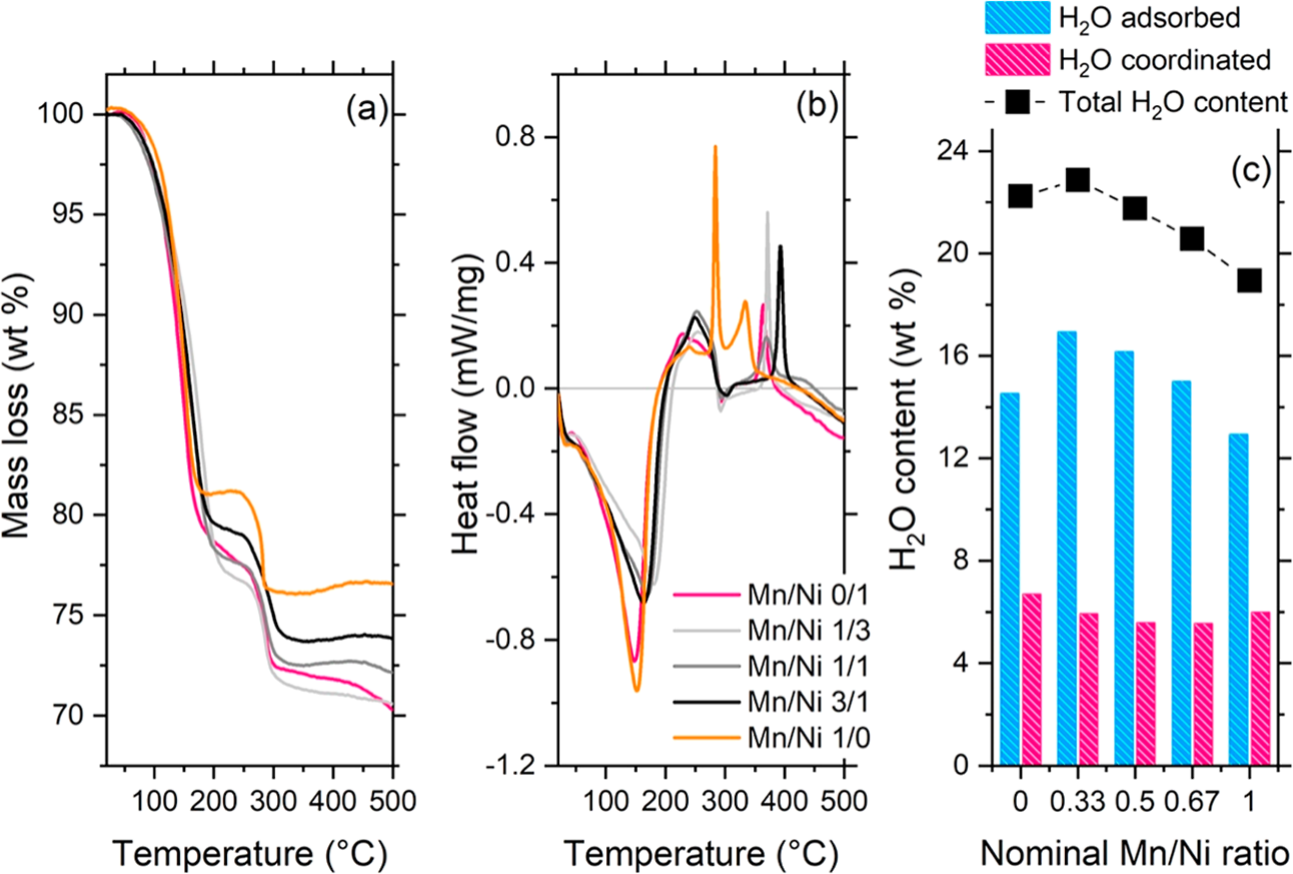
Thermal analyses of the samples under investigation: (a) thermal gravimetry and (b) differential scanning calorimetry (exo up). (c) Water content (mass percentage) as retrieved from the thermal analyses plotted vs the nominal Mn/Ni ratio (black markers: total water content; blue bars: adsorbed water content; pink bars: coordinated water content).

**Table 2:** Water Content (Weight %) as Retrieved from TGA

sample label	nominal Mn/Ni ratio	total H_2_O content (weight %)	adsorbed water (weight %)	coordinated water (weight %)
T3	0/1	22.23	14.53	6.70
T8	1/3	22.87	16.94	5.93
T6	1/1	21.75	16.16	5.59
T7	3/1	20.56	15.0	5.56
T5	1/0	18.94	12.94	6.0

Binary analogues displayed the lowest content of adsorbed water, while the ternary analogues showed a linear decrease in adsorbed water associated with the increase in manganese incorporated in the structure.

DSC measurements (Figure 3b) identified clear differences in the behavior of binary and ternary analogues: a symmetric exothermic peak centered around 150 °C and associated with the overall water loss was observed for the binary analogues, while an asymmetric peak at higher temperatures (about 180 °C for the sample with a Mn/Ni ratio 1/3, 170 °C for a Mn/Ni ratio of 1/1, and 163 °C for a Mn/Ni ratio of 3/1) was recorded for the ternary analogues. For these samples, the higher the manganese content, the lower the temperature of the overall process of water removal.

Material composition was investigated by means of EDX (Figure S2 in the Supporting Information), which allowed us to determine the metal content and hence the stoichiometry of the analogues. A summary of the results, including the empirical formula of the PBAs, is reported in Table 3, while Figure 4 shows an overview of their composition, displaying the Ni/Fe and Mn/Fe ratio as a function of the nominal Mn/Ni composition (Figure 4b, respectively) and the number of coordinated water molecules. It should be noted that the amounts of manganese and nickel in the empirical formulas were determined by considering that the pristine [Fe­(CN)_6_]^3–^ unit undergoes charge exchange during the reactions, lowering the amount of iron in oxidation state 3+ in favor of its partial reduction to Fe^2+^. This phenomenon is well acknowledged in the PBA literature and clearly visible in the FTIR spectra of the analogues, where the stretching mode of the cyanide ligand moves from a singlet in K_3_[Fe­(CN)_6_], to a deformed singlet with a tail toward lower energies in Prussian blue, to a doublet in binary Prussian blue analogues, due to the coordination of C=N with either Fe^II^ or Fe^III^.^7,19^

**Table 3:** PBA Composition and Empirical Formulas, as Retrieved by Combining the Information Obtained from EDX and Thermal Analyses

Mn/Ni ratio	sample label	K (at %)	Fe (at %)	Mn (at %)	Ni (at %)	formula	experimental Mn/Ni ratio
0/1	T3	2.12	11.4	0	17.4	K_0.38_Ni_2.92_[Fe(CN)_6_] _2_ × 8.98 H_2_O	0
1/3	T8	2.53	11.1	2.67	13.0	K_0.46_Mn_0.48_Ni_2.34_[Fe(CN)_6_] _2_ × 7.39 H_2_O	0.21
1/1	T6	1.45	7.00	3.70	5.30	K_0.32_Mn_1.08_Ni_1.62_[Fe(CN)_6_]_2_ × 6.74 H_2_O	0.67
3/1	T7	2.60	11.47	9.70	6.57	K_0.46_Mn_1.68_Ni_1.14_[Fe(CN)_6_] _2_ × 6.45 H_2_O	1.46
1/0	T5	1.10	4.53	6.53	0	K_0.48_Mn_2.88_ [Fe(CN)_6_] _2_ × 5.86 H_2_O	∞

**Figure 4:**
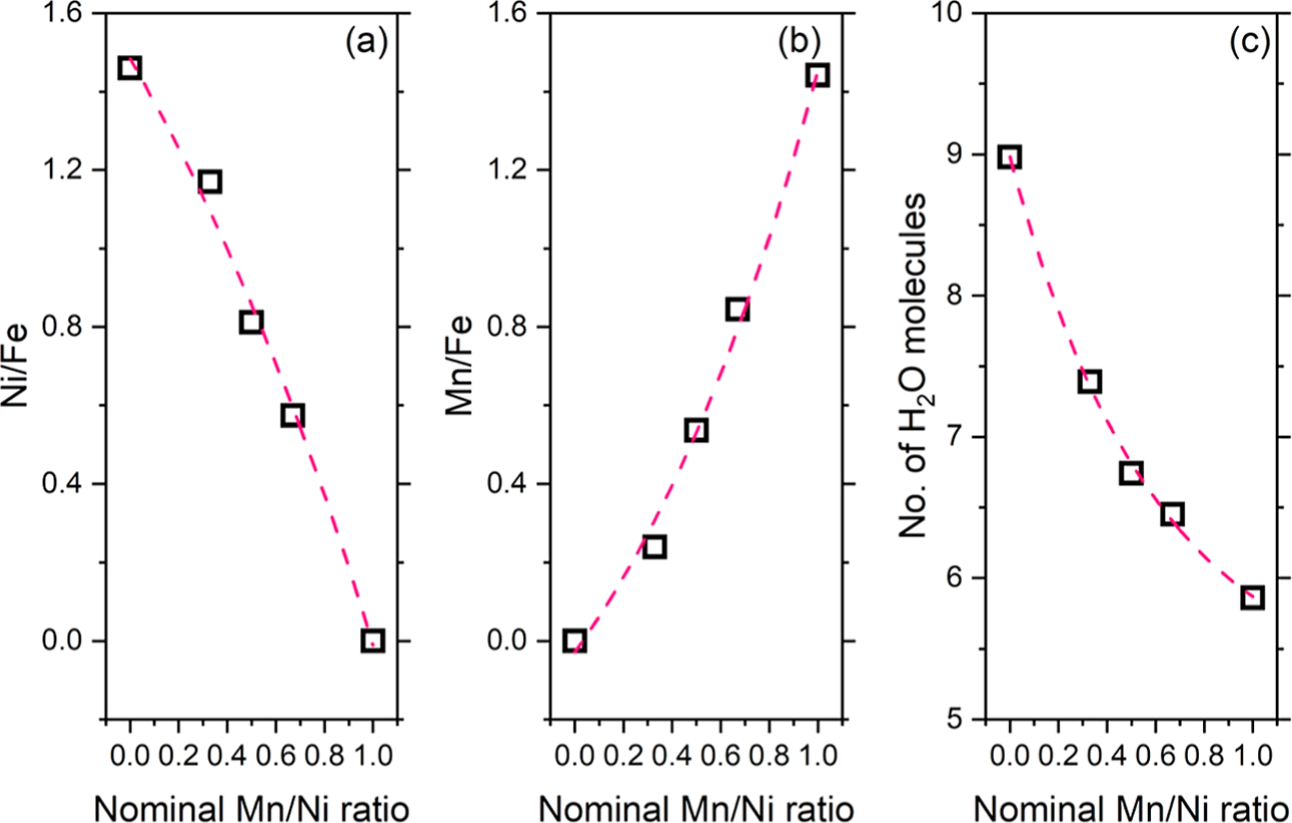
Compositional overview of the materials under investigation, as retrieved from SEM–EDX analyses and thermal analyses. Markers are experimental points; lines are fitting curves.

We extracted the ratio Fe^III^/Fe^II^ from the deconvolution of the C=N stretching mode in the FTIR spectra of the analogues, as reported in the pertaining section below. We then preferred the notation Fe^III^ and Fe^II^, i.e., with Roman numerals, to the use of the Arabic numerals 3+ and 2+ to follow the IUPAC nomenclature in coordination chemistry.

The Ni/Fe ratio showed an exponential decreasing trend with the nominal Mn/Ni ratio, while the Mn/Fe ratio displayed the opposite behavior: these findings account for the success of the synthetic approach, which is confirmed as a cheap, fast, and straightforward strategy to finely tune PBA composition. It is interesting to note that the amount of nickel in the ternary analogues was found to be higher than the nominal one: this observation is in agreement with the general acknowledgment that the equilibrium constants for the formation of metal complexes with nitrogen-containing ligands, considering the bivalent ions in the first transition series, are higher for Ni^2+^ than for Mn^2+^.^16^ This thermodynamic trend is then reflected in the experimental material composition.

The number of coordinated water molecules, calculated by merging the compositional information retrieved by EDX and TGA, was found to decrease exponentially with the nominal Mn/Ni ratio. These molecules act as ligands to coordinate the transition-metal ions in sites with vacancies of the [Fe­(CN)_6_] unit. The observed trend is explained based on the inertness toward water substitution shown by the Ni^2+^ aquo complex.

Concerning the amount of potassium, which did not show a clear trend with the nominal Mn/Ni ratio, opposite to the other metals, our interpretation of the experimental outcomes is as follows. We assumed that all nickel ions are in the oxidation state +2. This assumption is reasonable, due to the remarkably high standard redox potential for the semireaction Ni^3+^ → Ni ^2+^ (1.31 V vs. SHE for Ni­(OH)_3_ + e^–^ → Ni­(OH)_2_) and the low stability of its compounds.^20^ On the other hand, manganese can exist in different oxidation states and is prone to redox reactions also in the solid state.^21^

This makes it possible for the occurrence of charge exchange with the hexacyanoferrate unit, and potassium would then ensure electrical neutrality in the structure.

### 3.2 FTIR Analysis

Figure 5 shows the FTIR spectra of the prepared samples, highlighting the zone pertaining to the stretching of the cyanide ligand. Binary PBAs, largely studied in the literature through infrared spectroscopy, usually show two signals in this zone, ascribed to the stretching of the ligand when bound to Fe^III^ (higher wavenumbers) and to Fe^II^ (lower wavenumbers). In some cases, other minor signals are also detected, associated with the non-fully stoichiometric nature of the compounds.

**Figure 5:**
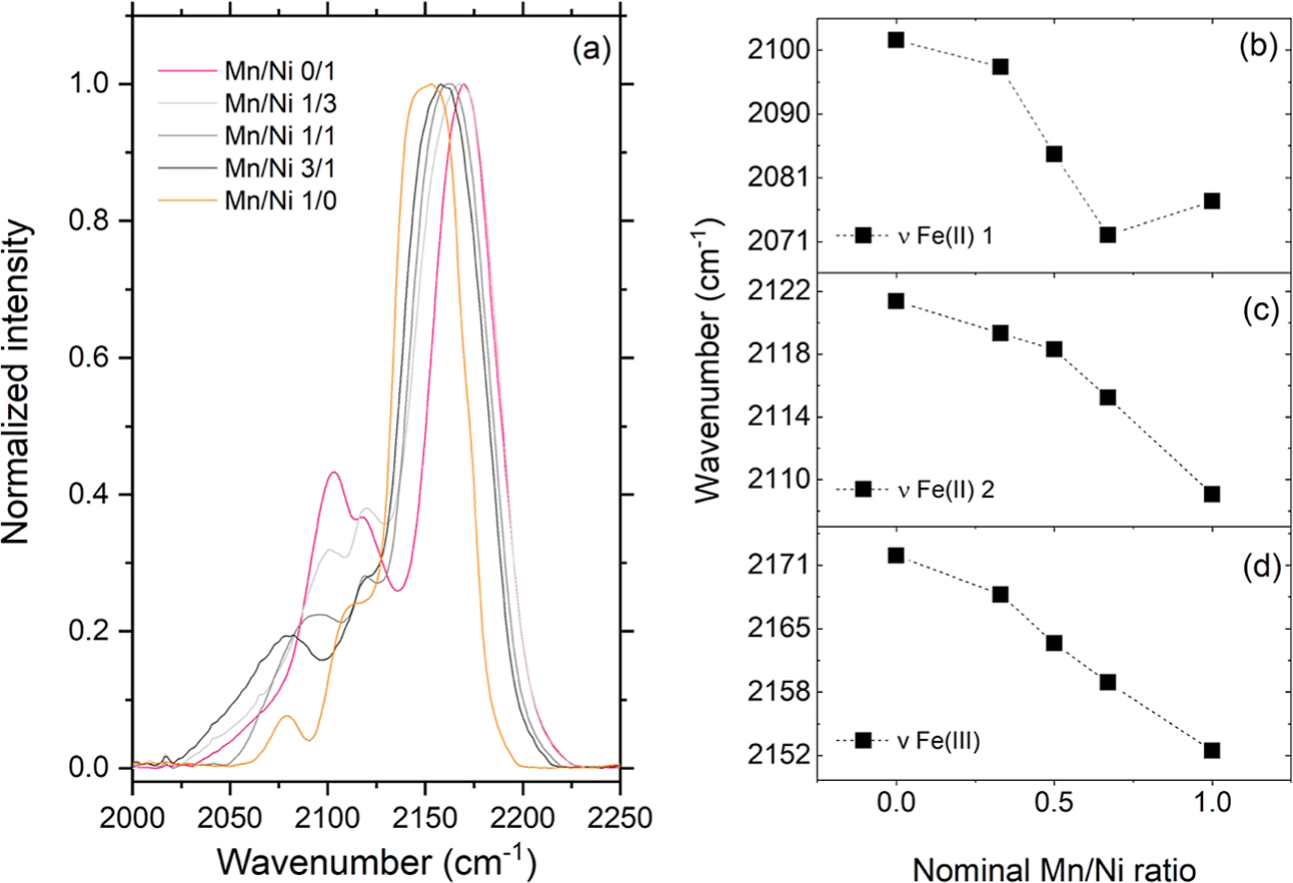
FTIR analysis of the samples under investigation. (a) Details of the CN stretching region. (b–d) Energy of the Fe–CN bond plotted as wavenumber as a function of the nominal Mn/Ni ratio.

The intense peak centered above 2150 cm^–1^ is ascribed to the stretching of the C=N ligand bound to Fe^III^. The energy associated with this stretching was observed to move to lower values as a function of sample composition: the higher the Mn amount, the lower the energy, with a difference of more than 20 cm^–1^ (about 2.48 meV) between the two binary analogues (Figure 5d). This finding well correlates with what was found in terms of lattice parameter from the XRD analysis: an increased amount of manganese induces a higher lattice constant, rendering the triple bond in the C=N ligand looser. From a coordination chemistry perspective, the trend can be read as an average decrease in the back-bonding from the iron ions induced by manganese (featuring a lower effective nuclear charge compared to nickel); i.e., the higher the amount of manganese, the lower the charge density donated from the iron ions to the π-antibonding orbitals of the cyanide ligand.

The shift of the energy of the C=N stretching bond correlating with PBA composition has been previously reported in the literature.^22,23^ Goberna-Ferrón and co-workers^22^ have, for instance, reported decreasing energies for the C=N stretching in binary hexacyanoferrate analogues M_*x*_[Fe­(CN)­6]_*y*_ (decreasing order for M = Cu, Ni, Co, Mn), which well correlates with electronegativity of the second metal M, confirming the analogy with metal complexes: the higher the electronegativity, the stronger the C=N bond (diminished π-back-bonding).

The peaks centered at lower wavenumbers (between 2100 and 2070 cm^–1^) are due to the C=N stretching when the ligand is bound to Fe^II^ (reduction of ferricyanide to ferrocyanide), and also in this case, significant changes in the strength of the C=N bond were observed with the material composition. In several cases reported in the literature for binary PBAs, a main peak in this region is accompanied by minor signals, attributed to the presence of metals in different oxidation states and to C=N linkages and/or C=N surface linkages.^19^ In our investigation, instead, we observed a clear splitting of the signal Fe^II^–C=N–M^n+^ in a doublet (whose components are labeled as 1 and 2 in Figure 5b,c), without any contribution from smaller vibrations (Figure 5a).

The peak between 2100 and 2120 cm^–1^ is not easily attributable: previous studies on binary PBAs based on cobalt hexacyanoferrates ascribed them to M^II^-Fe^II^ linkages, surface nonbridging C=N and residual M^III^-Fe^II^ linkages.^24^ In the present case, we observed the same trend between the decrease in the wavenumber of this signal and the amount of coordinated water molecules present in the structure. This induced us to identify a correlation of these two features: the higher the amount of water molecules, the stronger the C=N bond. Once again, this finding well aligns with the increase in the lattice parameter retrieved from the XRD analysis.

Finally, the signal at lower energy (2100–2070 cm^–1^): at present, we cannot assign this vibrational mode more precisely than saying that it pertains to the cyanide ligand bond to Fe^II^.

This analysis was confirmed by subjecting the samples to 4 h of irradiation with simulated solar light (calibrated with a Si reference cell, AM 1.5G, 1000 W/m^2^), expecting an oxidation of Fe^II^ to Fe^III^ and a corresponding change in the spectral vibrational features. The results are reported in Figure S3 in the Supporting Information. In order to quantify the oxidation process, the ratio Fe^III^/Fe^II^ was evaluated before and after sunlight exposure by fitting and integrating the corresponding infrared peaks (fitting shown in Figure S4 in the Supporting Information and pertaining data shown in Table S1 in the Supporting Information). In all samples but the binary nickel analogue and the one featuring Mn/Ni ratio equal to 1/3, the ratio between the Fe^III^–C=N (wavenumbers >2150 cm^–1^) and Fe^II^–C=N (wavenumbers <2120 cm^–1^) signals increased upon light exposure, confirming that (i) both vibrations below 2120 cm^–1^ pertain to the iron ion in a + II oxidation state and (ii) the presence of Ni^2+^, and its unruly nature toward redox processes, inhibits the oxidation of Fe^II^ to Fe^III^ through charge exchange between Fe^II^ and its neighbor transition metal ion connected through the cyanide ligand.

XRD analysis after irradiation with simulated solar light (reported in Figure S5 in the Supporting Information) showed the emergence of a peak around 21° for all the samples, except for the binary nickel analogue and the ternary sample with a Mn/Ni ratio of 3/1. This peak corresponds to the reflection from the (211) plane; this finding possibly indicates a phase transition from an fcc to a bcc lattice. The analysis of the unit cell size (shown in Figure S3b in the Supporting Information) revealed a significant change in the cell parameter only for the binary manganese sample.

Previous papers have shown that irradiation of PBAs can result in a modification of the crystal lattice, typically in a modification of the cell parameter.^15,25^ This modification, due to the mentioned charge transfer, is inhibited by the presence of Ni^2+^, which is not prone to redox processes.

### 3.3 Diffuse Reflectance Spectroscopy Analysis

Figure 6 reports the diffuse reflectance spectra of the prepared Prussian blue analogues and potassium hexacyanoferrate, used as a precursor, for comparison purposes. Almost identical spectral features were observed for the ternary analogues (three main reflectance minima centered around 24,450 cm^–1^, 32,680 cm^–1^, and 43,000 cm^–1^). The binary analogues displayed a slightly different behavior: the binary nickel analogue also showed three minima centered at slightly different energies (24,450, 33,445, and 44,444 cm^–1^), while the manganese binary analogue displayed only two minima (24,450 cm^–1^, with an unresolved peak at lower wavenumbers, 20,790 cm^–1^, and 29,940 cm^–1^).

**Figure 6:**
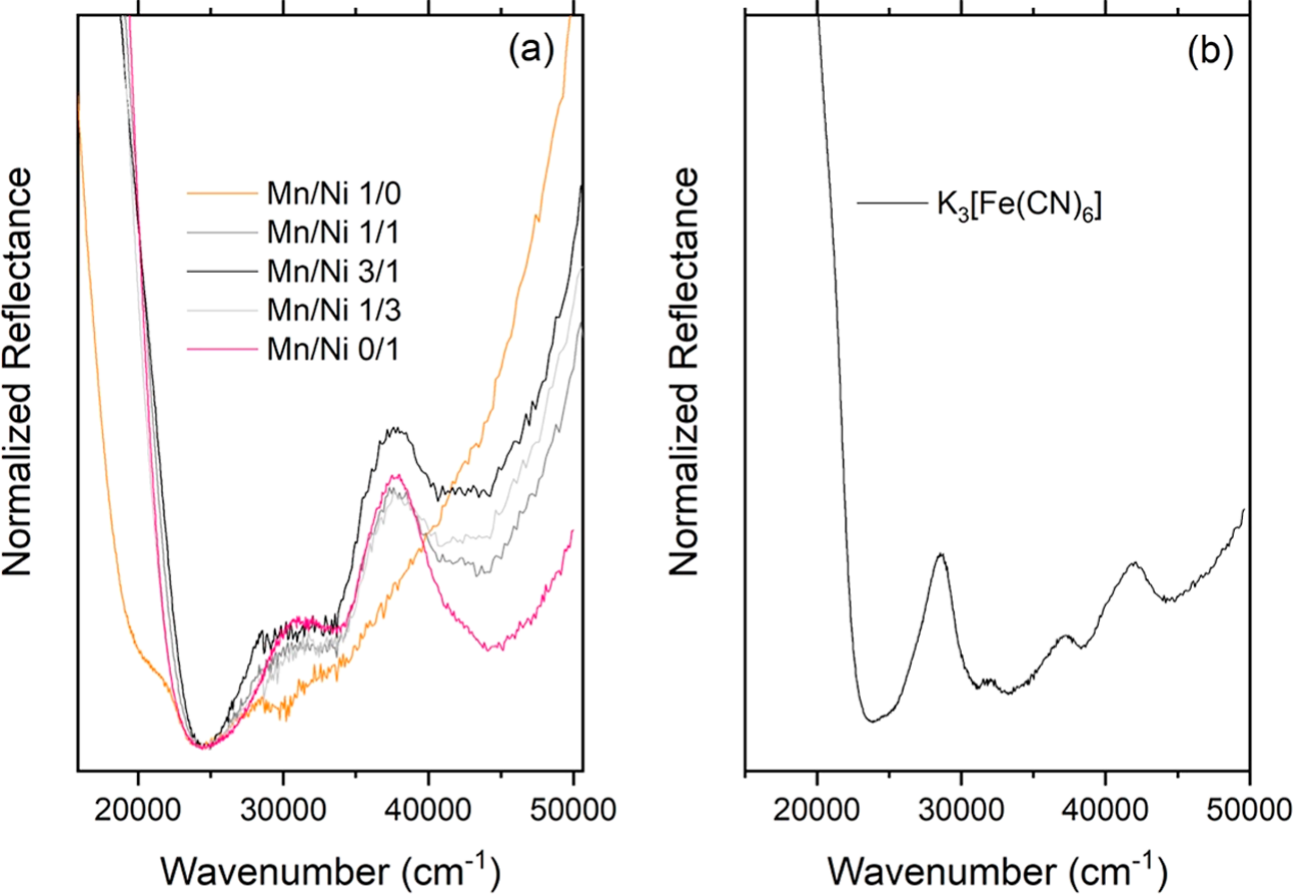
Diffuse reflectance spectra of (a) PBAs and (b) the hexacyanoferrate precursor.

The nature of these minima is interpreted in the light of the spectral characteristics of the precursor K_3_[Fe­(CN)_6_], analyzed by analogy with the outcomes of coordination chemistry: this compound features four main minima, centered at 23 952 cm^–1^ (attributed to ligand-to-metal charge transfer—LMCT), 33,222 cm^–1^ (crystal field stabilization energy, the so-called octahedral delta, Δ_0_, in the metal complex), 38,389 cm^–1^ (not attributed), and 44,444 cm^–1^ (t6 → t5e absorption).^16,26,27^

It is then interesting to remark the following: (i) the LMCT occurs at the same energy in all the analogues, with an additional contribution in the Mn binary compound; (ii) the octahedral splitting energy is the same for all the ternary materials, supporting the same band structure in all of them, and it is slightly reduced compared to that observed in the precursor, while a significant difference in energy is observed in the Mn and Ni binary analogues (diminished in the former and increased in the latter, due to the lower crystal field stabilization energy for a d^5^ transition metal ion); (iii) the signal pertaining to the lowest crystal field transition is completely quenched in the Mn analogue, indicating a different orbital configuration for this compound.

### 3.4 XPS Analysis

The elemental composition of the samples was also investigated by XPS, accessing the outermost layers (<10 nm) of the materials, to provide information on the surface chemical composition, including the oxidation state of the components. The obtained results are shown in Table S1 in the Supporting Information (and displayed in Figures S5–S8), where it can be seen that the samples were characterized by the presence of C, N, O, K, Fe, Ni, and Mn. The XPS quantitative analysis confirmed the results displayed in Table 3, in particular, as for the Mn/Ni atomic ratios, although a slight excess of Fe and Ni on the surface was registered. Figure 7 shows the comparison of Fe 2p signals, characterized by the typical doublet Fe 2p_3/2_-Fe 2p_1/2_, due to the spin–orbit splitting. The peak-fitting analysis evidenced the presence of two Fe 2p_3/2_ components in the manganese binary analogue, positioned at BE = 710.2 and 713.1 eV, assigned to [Fe^III^(CN)_6_]^3–^ and its shakeup satellite,^28^ respectively. Interestingly, the remaining samples were characterized by an additional component localized at BE = 707.5 ÷ 707.9 eV, at first glance, which should be assigned to Fe in a metallic state. However, since there are no conditions to believe that a strong reduction of Fe occurred during the reaction, most likely, that component can be assigned to [Fe^III^(CN)_5_H_2_O]^2–^, as suggested in ref 28. The presence of the cyano group was confirmed in C 1s and N 1s signals, although the C 1s signal was affected by overlap with the adventitious carbon. Moreover, in the N 1s signal, a component at BE ∼ 401.2 eV was also identified, due to the π–π* transition in the cyanide ligand.^29^ Concerning Mn 2p and Ni 2p signals, the obtained value indicated that they were included in the PBA structure. However, the Mn 2p_3/2_ peak was positioned at BE = 642.0 eV, an intermediate value between Mn^III^ and Mn^IV^,^28^ while the Ni 2p_3/2_ was positioned at BE = 856.4 eV, which was assigned to Ni^2+^.^30^

**Figure 7:**
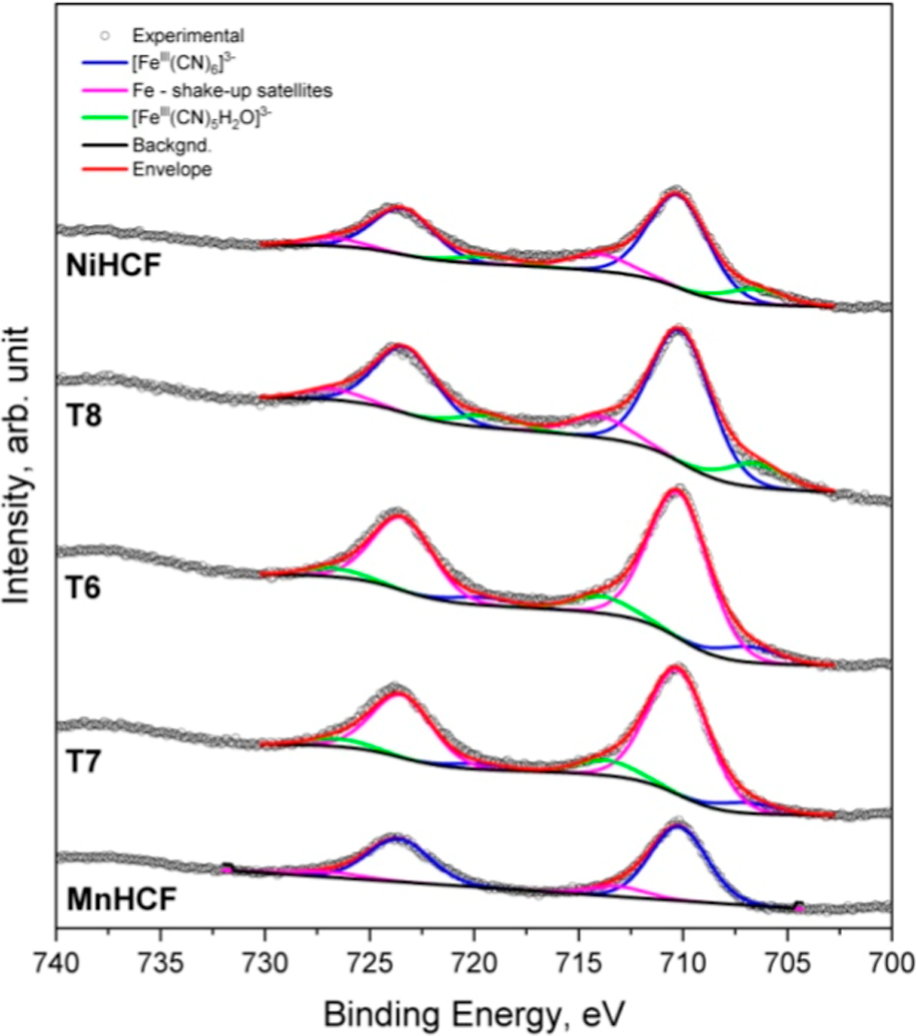
Comparison of the XPS Fe 2p signal of all the investigated samples. NiHCF and MnHCF indicate the nickel and manganese binary analogues, respectively.

### 3.5 Electrochemical Characterization

Cyclic voltammetry was applied to the prepared samples to investigate their skill in responding to a bias stimulus by promoting internal redox reactions and the intercalation of alkaline cations.

Plain differences in the cyclic voltammograms were observed (Figure 8 shows the CV curve recorded in Na_2_SO_4_ at a scan rate of 100 mV/s). A complete overview of the CV measurements in both Na_2_SO_4_ and K_2_SO_4_ is reported in Figures S10 and S11 in the Supporting Information.

**Figure 8:**
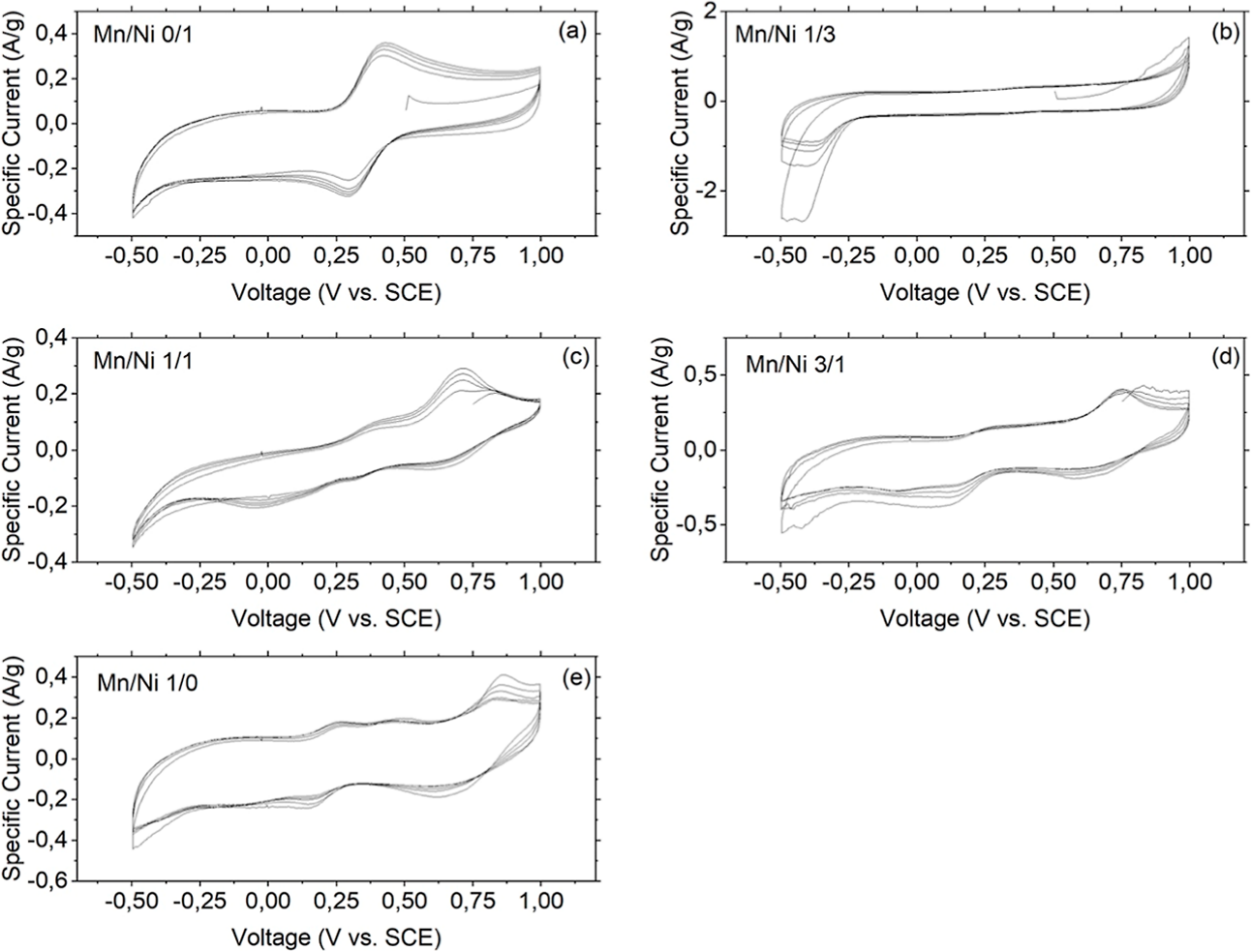
Cyclic voltammograms for all the samples under investigation in Na_2_SO_4_ (scan rate = 100 mV/s). (a–e) Increasing amount of manganese.

The binary analogue with nickel (Figure 8a) displays a pair of redox peaks (centered at about 428 mV and 293 mV for the anodic and cathodic processes, respectively), which are ascribed to the redox couple Fe­(III)/Fe­(II). As expected, no redox process associated with nickel was observed in the potential range explored.

Insertion of manganese at a Mn/Ni ratio of 1/3 (Figure 8b) resulted in an almost flat curve and a higher current; a closer inspection revealed small shoulders in both scanning directions, centered at the same voltages as those observed in the analysis of the nickel binary analogues, attributable to the same redox couple, whose activity was however hidden by the higher amount of current (about an order of magnitude compared with that recorded for the nickel binary analogue).

Further manganese addition resulted in more complex redox patterns (Figure 8c–e), where the signature of redox processes undergone by manganese could be observed.^31,32^ The peaks pertaining to the iron redox couple were still visible, but less pronounced.

Similar behaviors were found by changing the electrolyte to K_2_SO_4_ in terms of the positions of the redox peaks. Manganese-containing *t*-PBAs appear to be rather insensitive to the nature of the cation in the electrolyte (a different behavior compared to that observed in analogues with other transition metal ions, like cobalt).^33^ This finding was previously reported by Jayasri and Narayanan,^34^ who also observed similar trends in Na^+^- and K^+^-based electrolytes and highlighted the contradictory behavior compared to other PBAs, which are usually not very permeable to sodium ions. Their conclusion, which we agree with in the present investigation, is that the ion transportation in Mn-based PBAs is complex and cannot be accounted for on the basis of simple considerations on the interplay between cationic hydrated ionic radii and cavity sizes.

CV curves showed an increase in current associated with the increase in the scan rate, testifying to a good Nernstian behavior and reversibility in both electrolytes. The samples containing the higher amounts of Mn (Mn/Ni ratio 3/1 and 1/0) showed a certain instability in the voltammograms, in terms of the current associated with the redox processes, which was previously reported in the case of manganese hexacyanoferrate and is associated with a stabilization process of the electrodes.^35^

Manganese hexacyanoferrate is considered as a promising material for the field of electrochemical energy storage.^35^ We then evaluated the charge storage control mechanism through the *b* parameter, calculated by linearizing the following equation: *J* = *k*ν^*b*^, where *J* is the current observed in the CV curves for redox processes (anodic peaks were considered in this case), *v* is the scan rate, and *k* is a proportionality constant. When *b* = 0.5, the charge storage is controlled kinetically through diffusion, while the closer the *b* value is to 1, the more capacitive the mechanism of storing charge becomes.^36^

The results are reported in Figures S10 and S11 in the Supporting Information. Varying the amount of manganese had a relevant impact on the charge storage control mechanism. The contribution of capacitive control significantly increased with the amount of manganese, reaching values close to or equal to unity for Mn/Ni ratios of 3/1 and 1/0. The only exception to the linear increase in the *b* parameter was observed with the sample featuring a Mn/Ni 1/1, which displayed the lowest *b* value of the batch in Na_2_SO_4_ and the smallest *b* value of the Mn-containing samples in K_2_SO_4_.

Specific capacitance was also evaluated from the CV curves, according to the following equation: $C=\frac{Q}{\Delta V\times w}$ , where Q is the charge ( $Q=\frac{Area}{2\times \nu }$ , with ν being the scan rate), Δ*V* is the voltage window, and w is the weight of the active material.

Despite the charge storage control being almost completely capacitive for the samples with the highest amount of manganese, the best specific capacitance was found to belong to the binary nickel analogue (Table S2 in the Supporting Information). This specimen displayed an increase in the capacitance over the cycling, more marked in Na_2_SO_4_ (extended cyclic voltammograms for 200 cycles are shown for both electrolytes in Figures S11 and S12 in the Supporting Information).

Moreover, the capacitance retention (as retrieved from cycling the materials 200 times at 100 mV/s) featured by the manganese analogues was found to quickly decrease (Figure S14 in the Supporting Information). The cyclic voltammograms showed the progressive disappearance of redox signals in all of the Mn-containing analogues: this is possibly a sign of a structural change (previously observed in cobalt-containing PBAs).^7^ The electrochemical inertia of nickel, on the contrary, preserves the original features, ensuring stability.

We also investigated the galvanostatic charge–discharge behavior of the manganese-containing analogues in Na_2_SO_4_ (Figure S15 in the Supporting Information). Interestingly, we observed curves closely resembling those of an RC capacitor for all the analogues (Figure 9), irrespective of the constant current applied (full curves are displayed in Figure S14 in the Supporting Information) well fitted by an exponential equation of the kind: *V*=(Ae^x/τ^)+*V*_0_, where τ is the charging/discharging time constant (the values are reported in Table S3 in the Supporting Information).

**Figure 9:**
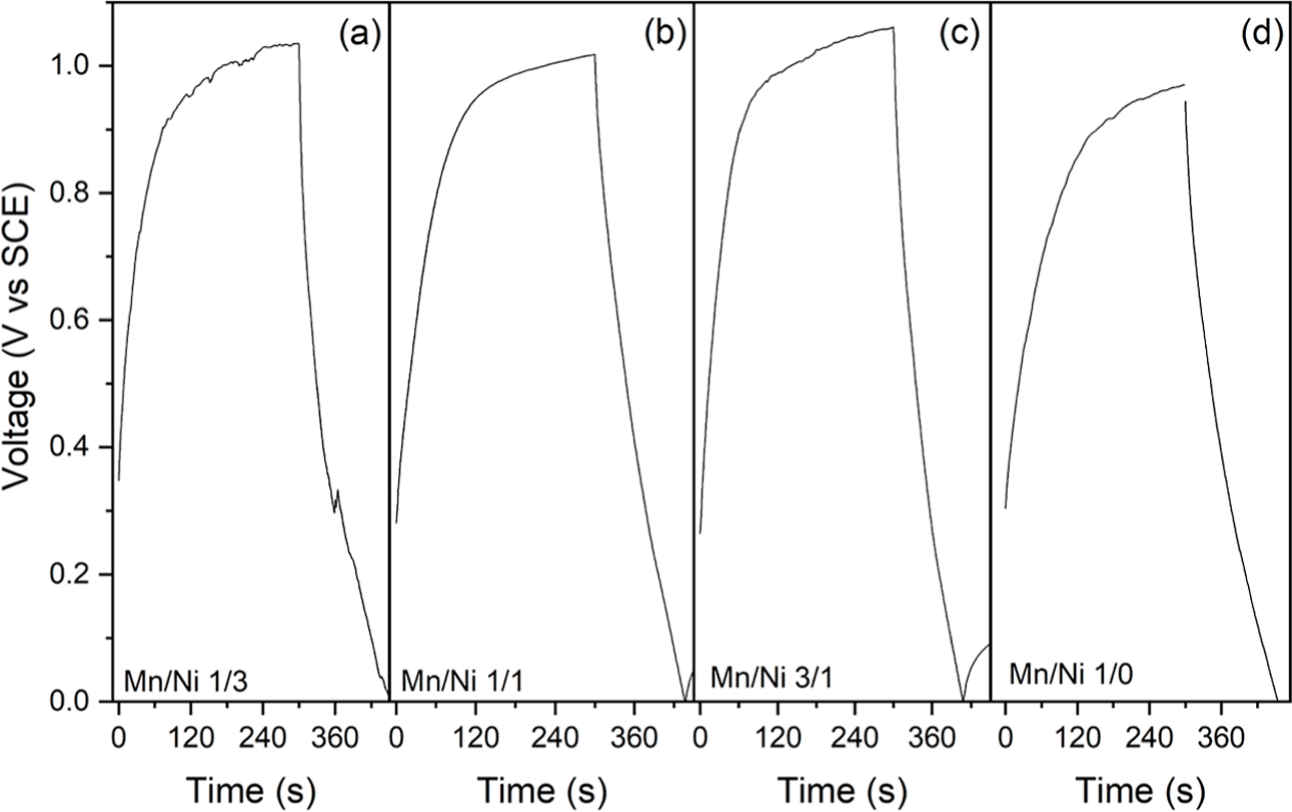
Galvanostatic charge–discharge curves (*J* = 25.75 mA/g) recorded for the manganese-containing analogues. (a–d) Increasing amount of manganese.

The asymmetry of the curves is a well-known phenomenon in porous supercapacitors, implying a nonideal behavior. This can have different origins, such as, for instance, internal resistance paths differing between charging and discharging. In this experiment, we have allowed a charging upon constant applied current (25.74 mA/g) for 300 s, during which all the analogues reached a voltage around 1 V vs SCE. In this potential range, CV showed more faradaic processes in the analogues featuring a Mn/Ni ratio of 1/1 and 1/0, which indeed are the materials displaying the longest time constants. This suggests the presence of activation barriers, to overcome the energy required, a slower kinetics.

Interestingly, the only analogue showing a voltage drop (26 mV) between charge and discharge was the manganese binary analogue (Figure 9d).

## 4 Conclusions

This work demonstrates that controlled cosubstitution of Ni^2+^ and Mn^2+^ in ternary Prussian blue analogues enables fine-tuning of their structural and functional properties. The Mn/Ni ratio dictates both lattice expansion and crystallite size, mirroring the interplay between ionic radii and ligand-exchange kinetics in solution. Increasing manganese content decreases the number of coordinated water molecules and weakens the Fe–CN bonding, resulting in a less defective, more open framework.

Upon sunlight irradiation, structural and spectroscopic analyses revealed that Ni^2+^ plays a crucial stabilizing role: while Mn-rich samples underwent lattice modification and partial phase transition associated with photoinduced Fe^2+^ → Fe^3+^ charge transfer, Ni-containing analogues maintained structural integrity and spectral stability.

Electrochemical measurements further confirmed this dual behavior—Ni-rich compositions offered superior capacitance retention and bias stability, whereas Mn-rich materials displayed enhanced pseudocapacitive response but reduced cycling durability.

Overall, these results illustrate how coordination chemistry principles govern both the photochemical and electrochemical behavior of ternary Ni–Mn–Fe Prussian blue analogues, offering a pathway for designing robust, multifunctional materials for photoelectrochemical and energy storage applications.

## Supplementary Material



## Notes

Result of XRD LeBail refinement; EDX analysis; details of the CN stretching region and peak fitting for the CN stretching; Fe­(III)/Fe­(II) ratio as calculated from the fitting of FTIR CN vibration mode; XRD patterns of the materials after 4 h of irradiation with simulated solar light; XPS results; CV and electrochemical parameters; and capacitance retention and galvanostatic charge/discharge analysis (PDF)
