# Supplementary material for: Synthesis and Characterization of Ternary Manganese–Nickel–Iron Prussian Blue Analogues: Bridging Coordination Chemistry and Solid-State Physics
Source: ACS Omega. 2026 May 5;11(20):29992–30002. doi: 10.1021/acsomega.6c01289 (PMC13216981; doi:10.1021/acsomega.6c01289)
Supplement: Supplementary file 1 [file ao6c01289_si_001.pdf]

Supporting Information

Synthesis and characterization of ternary manganese-nickel-iron Prussian blue analogues: bridging coordination chemistry and solid state physics

Isabella Concina[a,*], Alessio Mezzi[b], Shujie You[a]

[a] Department of Engineering Sciences and Mathematics, Luleå University of Technology, Luleå Sweden

[b] CNR, Istituto per lo Studio dei Materiali Nanostrutturati, Strada Provinciale 35d, n. 9, 00010 Montelibretti (RM)

*Corresponding Author: isabella.concina@ltu.se

(a) Sample T5 (Mn/Ni 1/0), R_{wp}=10.40%, R_{exp}=7.25%, a = 10.5163 ± 0.0001 Å

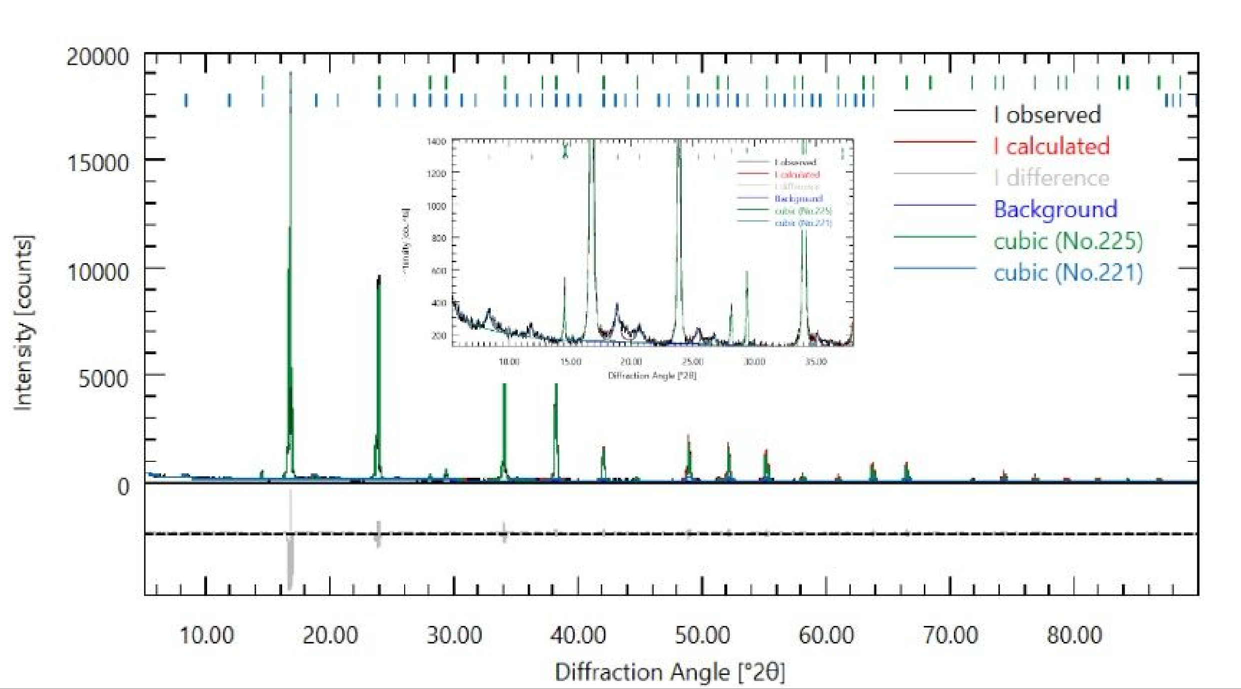

(b) Sample T7 (Mn/Ni 3/1), R_{wp}=8.86%, R_{exp}=5.26% a =10.3990 ± 0.0009 Å

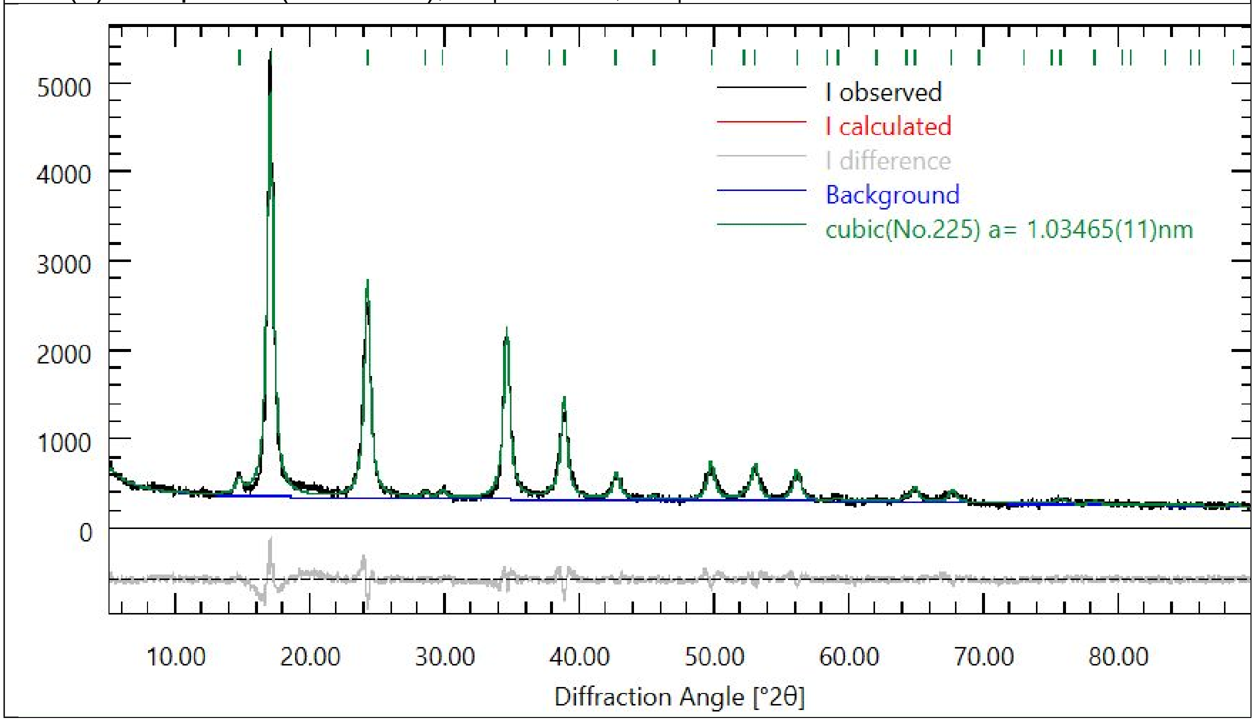

(c) Sample T6 (Mn/Ni 1/1), R_{wp}=10.40%, R_{exp}=7.25%, a =10.3465 ± 0.0011 Å

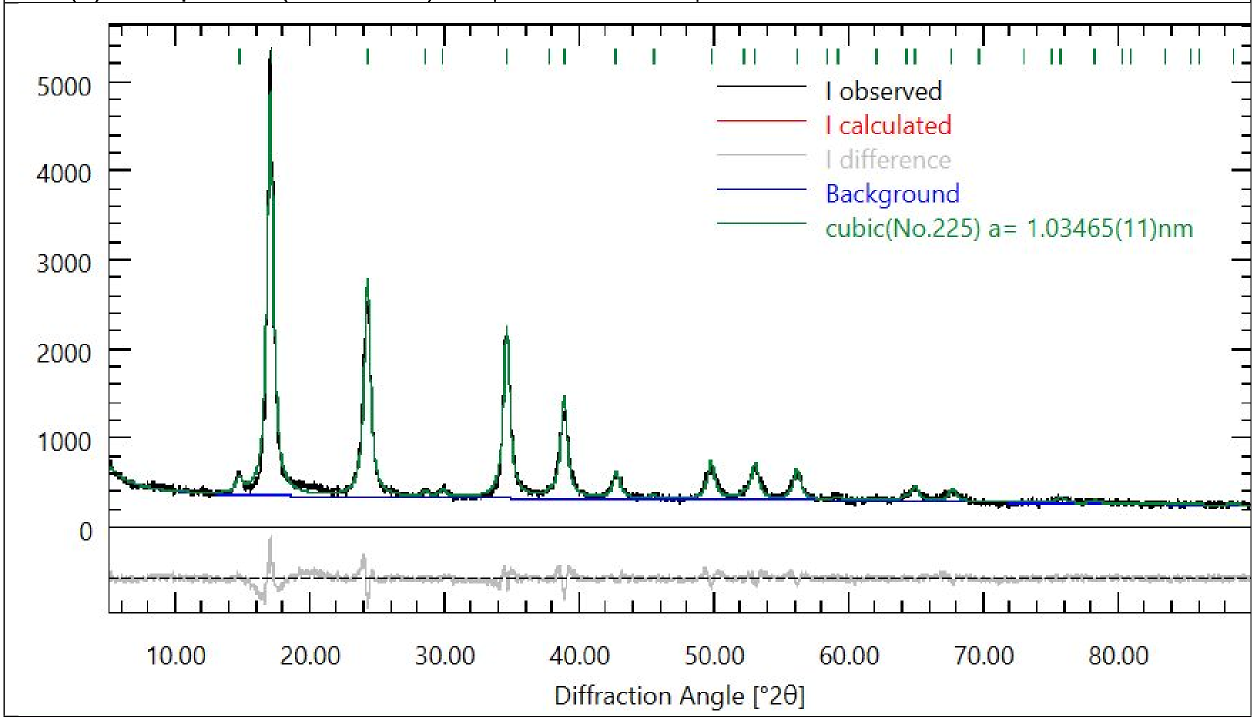

(d) Sample T8 (Mn/Ni 1/3), R_{wp}=7.07%, R_{exp}=4.07%, a =10.2834 ± 0.0013 Å

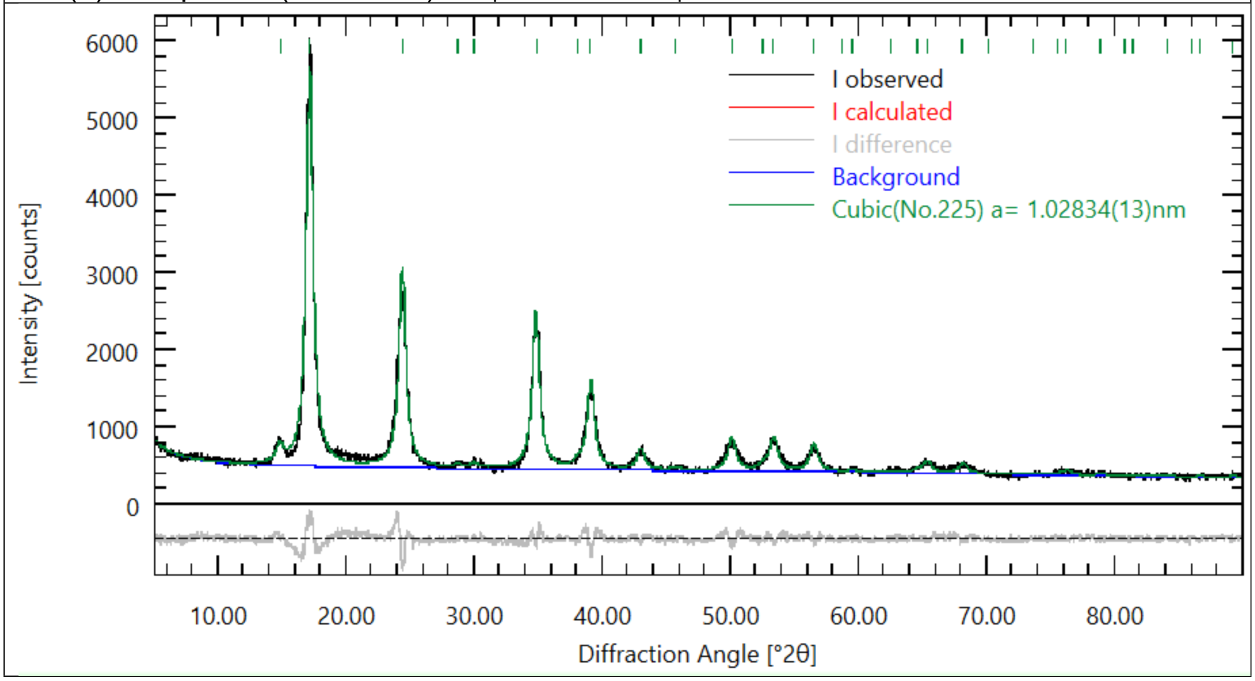

(e) Sample T3 (Mn/Ni 0/1), R_{wp}=6.55%, R_{exp}=4.45%, a =10.2322 ± 0.0017 Å

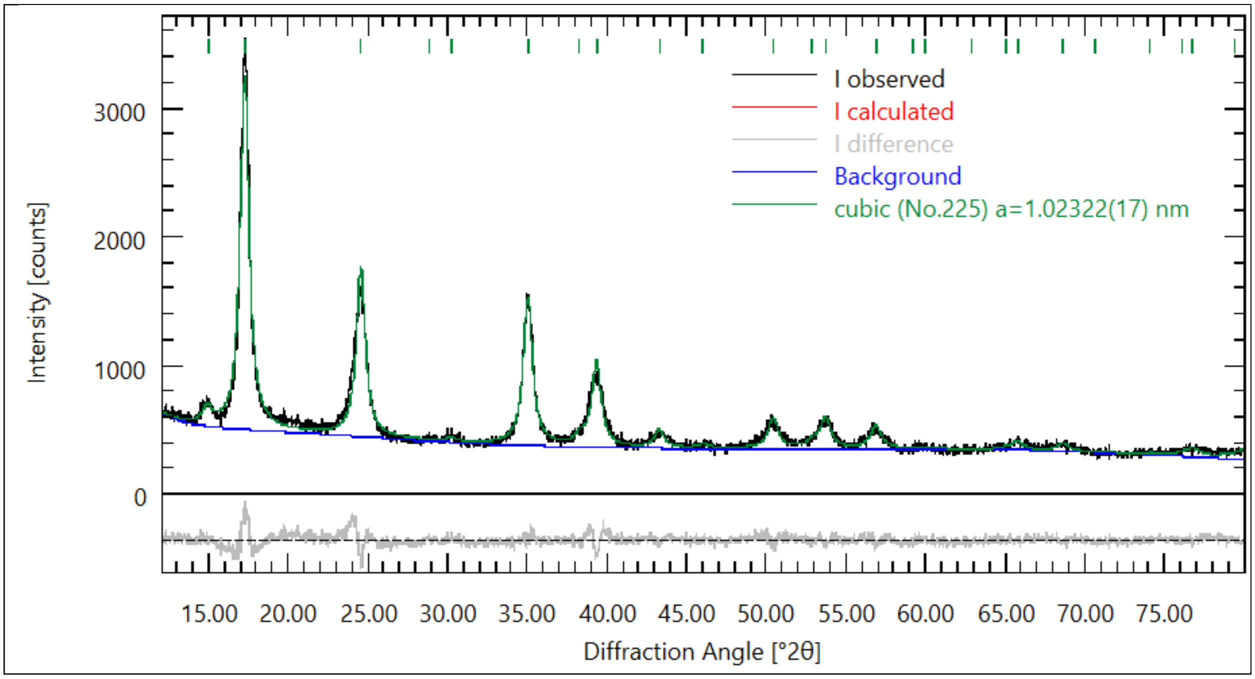

Figure S 1. Result of XRD LeBail refinement.

Sample lable: T5	Mn/Ni/Fe ratio:	2.90 / 0 / 2.00
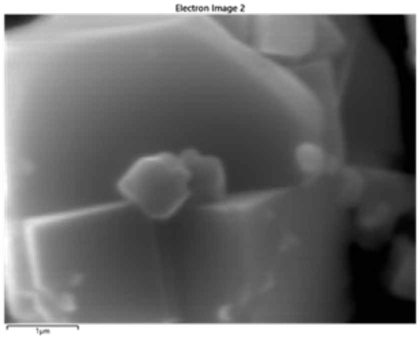	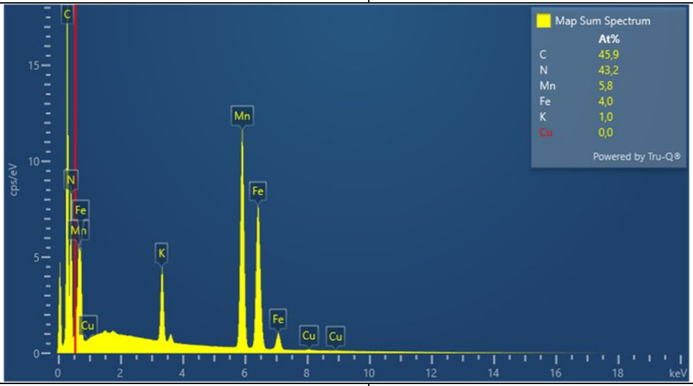	
Mn Kα1 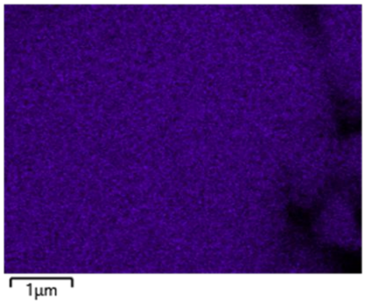	Fe Kα1 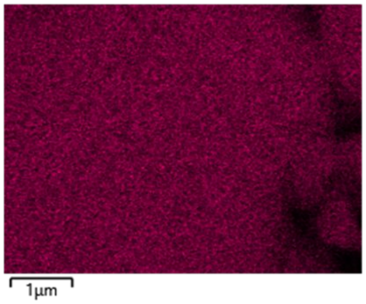	

Sample lable: T6	Mn/Ni/Fe ratio:	1.09 / 1.76 / 2.00
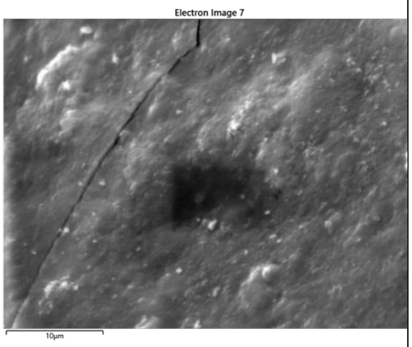	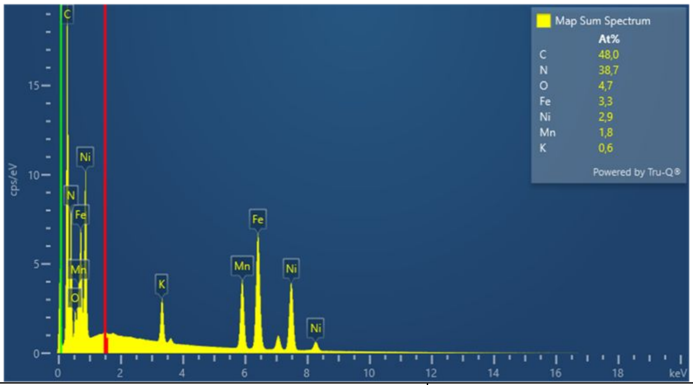	
Mn Kα1 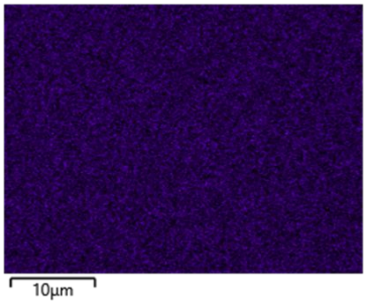	Ni Kα1 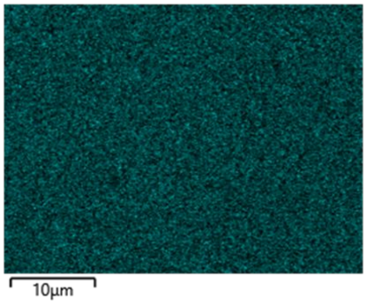	Fe Kα1 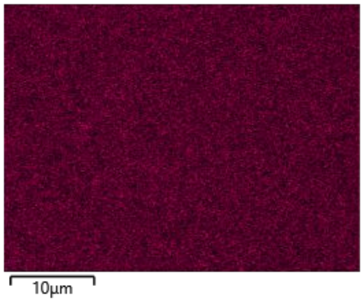

<table>
<tr><td>Sample lable: T7</td><td>Mn/Ni/Fe ratio:</td><td>1.70 / 1.15 / 2.00</td></tr>
<tr><td>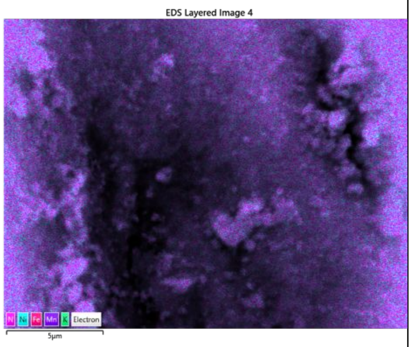
</td><td colspan="2">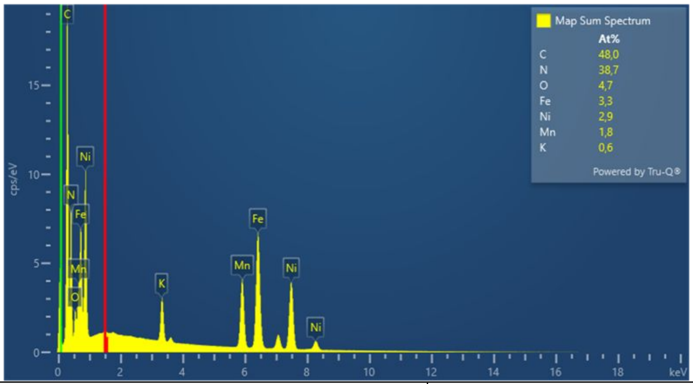
</td></tr>
<tr><td>Mn Kα1
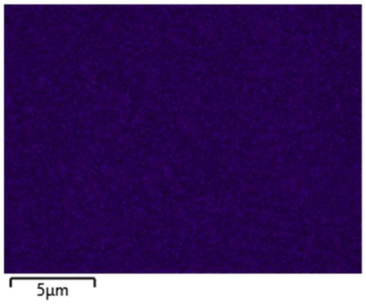
</td><td>Ni Kα1
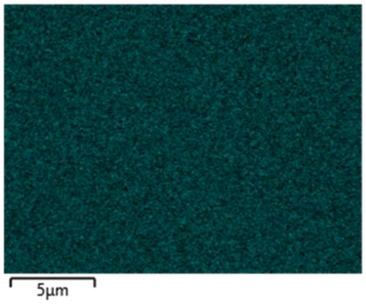
</td><td>Fe Kα1
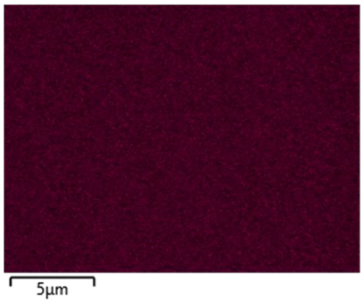
</td></tr>
</table>

<table>
<tr><td>Sample lable: T8</td><td>Mn/Ni/Fe ratio:</td><td>0.46 / 2.30 / 2.00</td></tr>
<tr><td>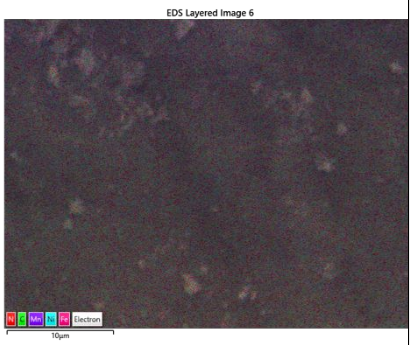
</td><td colspan="2">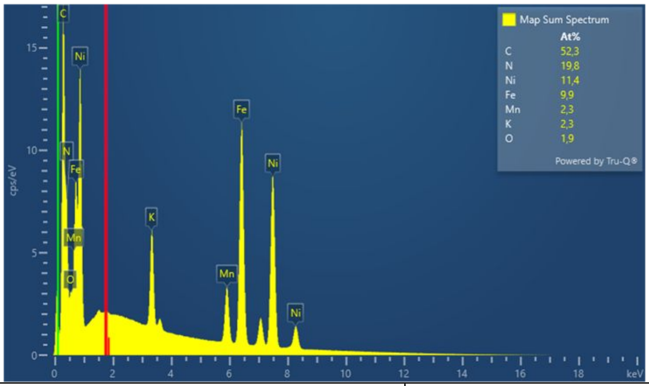
</td></tr>
<tr><td>Mn Kα1
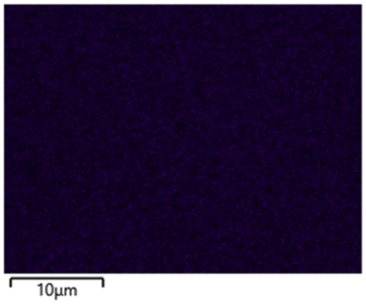
</td><td>Ni Kα1
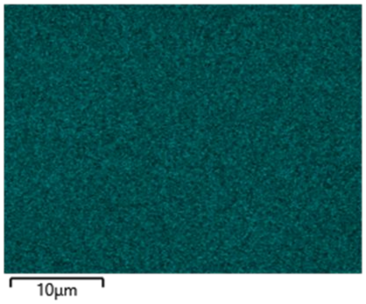
</td><td>Fe Kα1
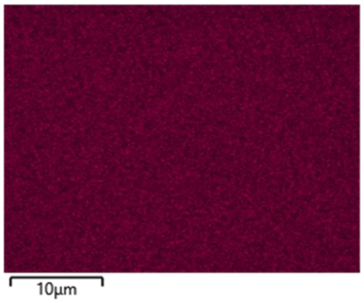
</td></tr>
</table>

<table>
<tr><td>Sample lable: T3</td><td>Mn/Ni/Fe ratio:</td><td>0 / 2.95 / 2.00</td></tr>
<tr><td>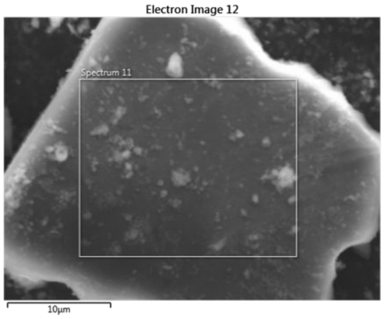
</td><td colspan="2">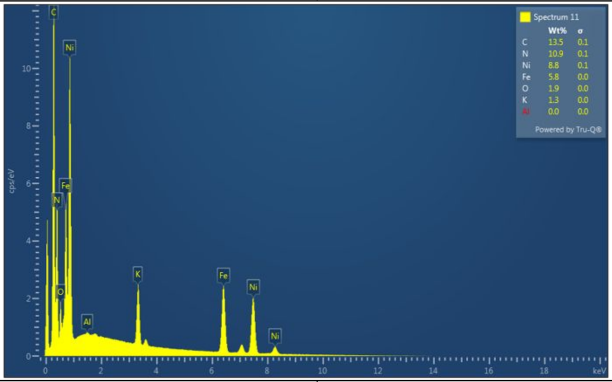
</td></tr>
<tr><td>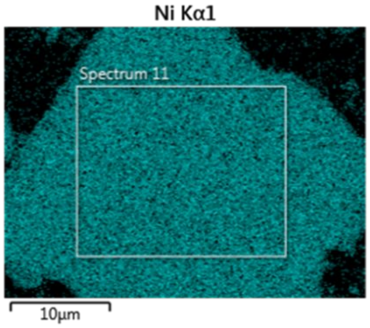
</td><td>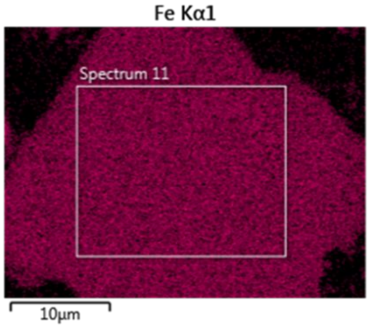
</td><td></td></tr>
</table>

Figure S 2. EDX analysis.

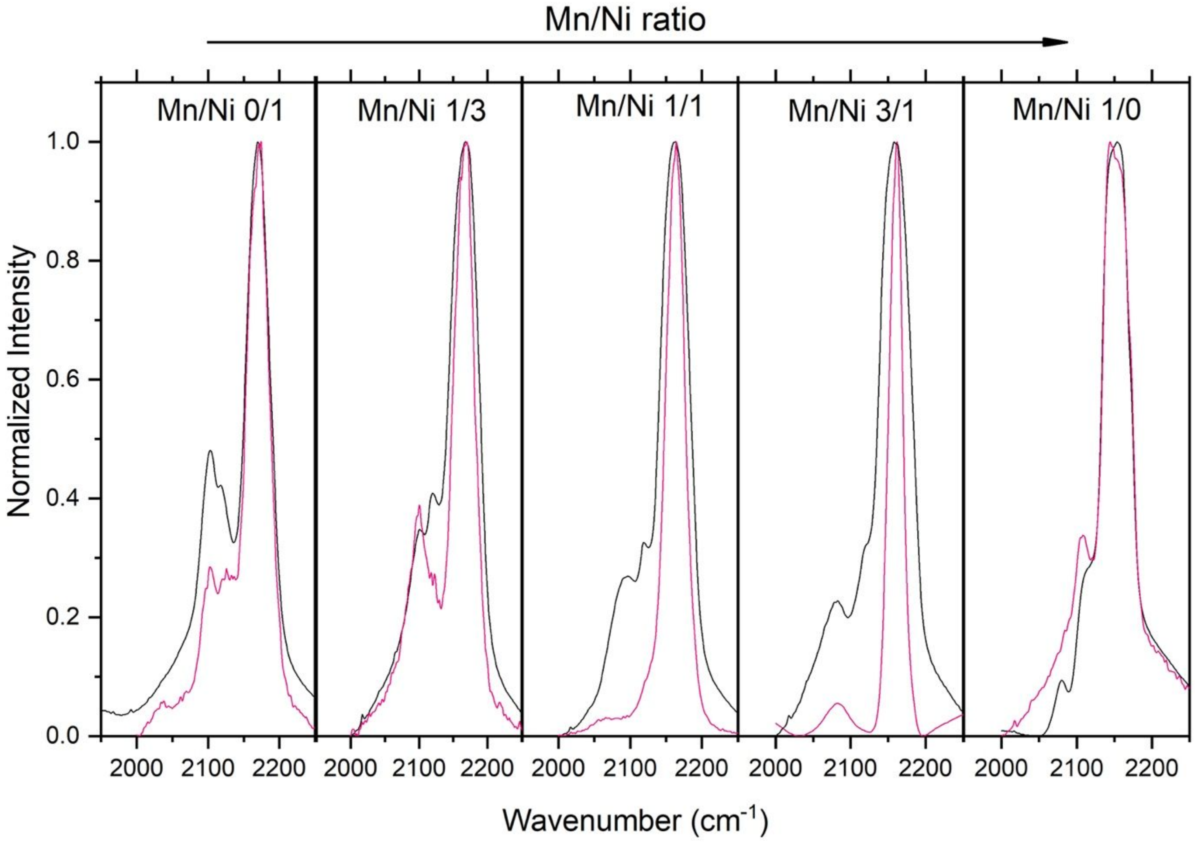

Figure S 3. Detail of the CN stretching region (normalized intensity) for the prepared samples (left to right: increasing Mn/Ni ratio). Black line: as prepared samples; pink line: samples irradiated with simulated solar light for 4 h.

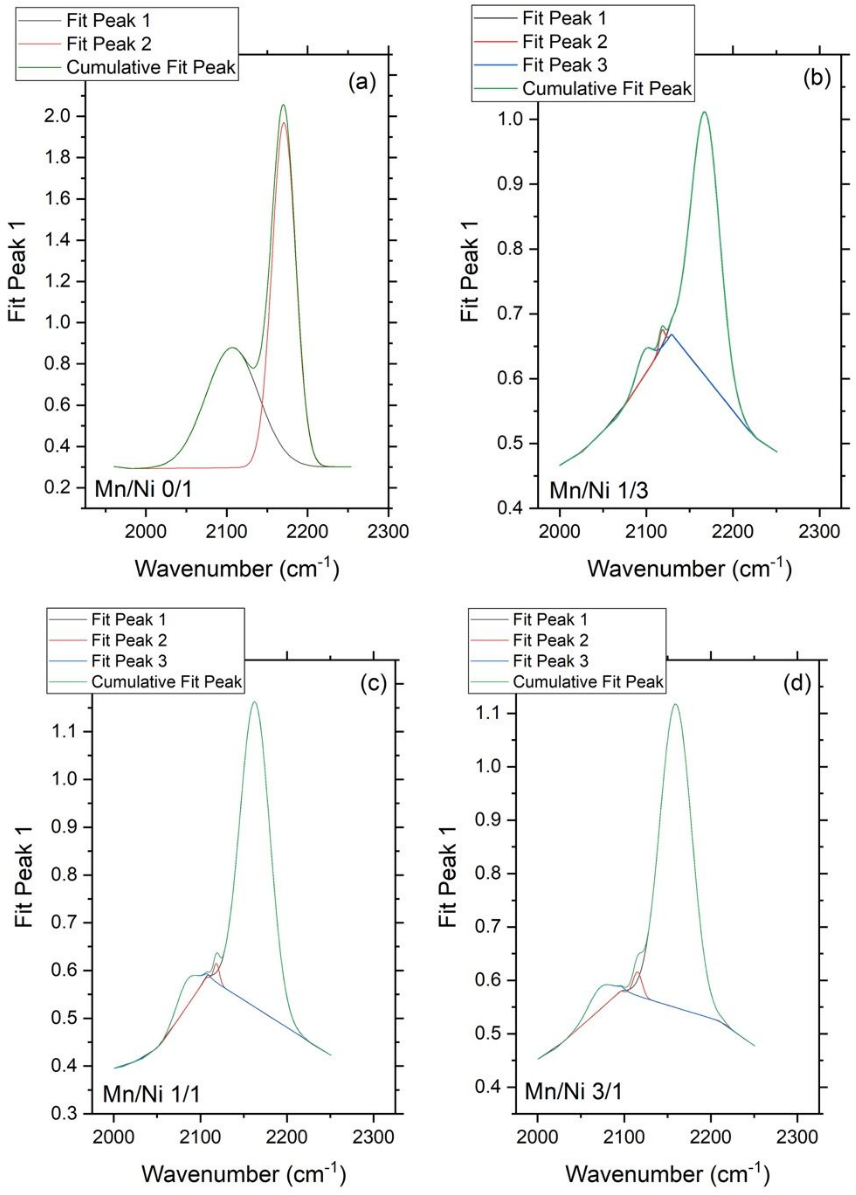

Fit Peak 1
Fit Peak 2
Cumulative Fit Peak
(a)
Mn/Ni 0/1
Fit Peak 1
Wavenumber (cm-1)
Fit Peak 1
Fit Peak 2
Fit Peak 3
Cumulative Fit Peak
(b)
Mn/Ni 1/3
Fit Peak 1
Wavenumber (cm-1)
Fit Peak 1
Fit Peak 2
Fit Peak 3
Cumulative Fit Peak
(c)
Mn/Ni 1/1
Fit Peak 1
Wavenumber (cm-1)
Fit Peak 1
Fit Peak 2
Fit Peak 3
Cumulative Fit Peak
(d)
Mn/Ni 3/1
Fit Peak 1
Wavenumber (cm-1)

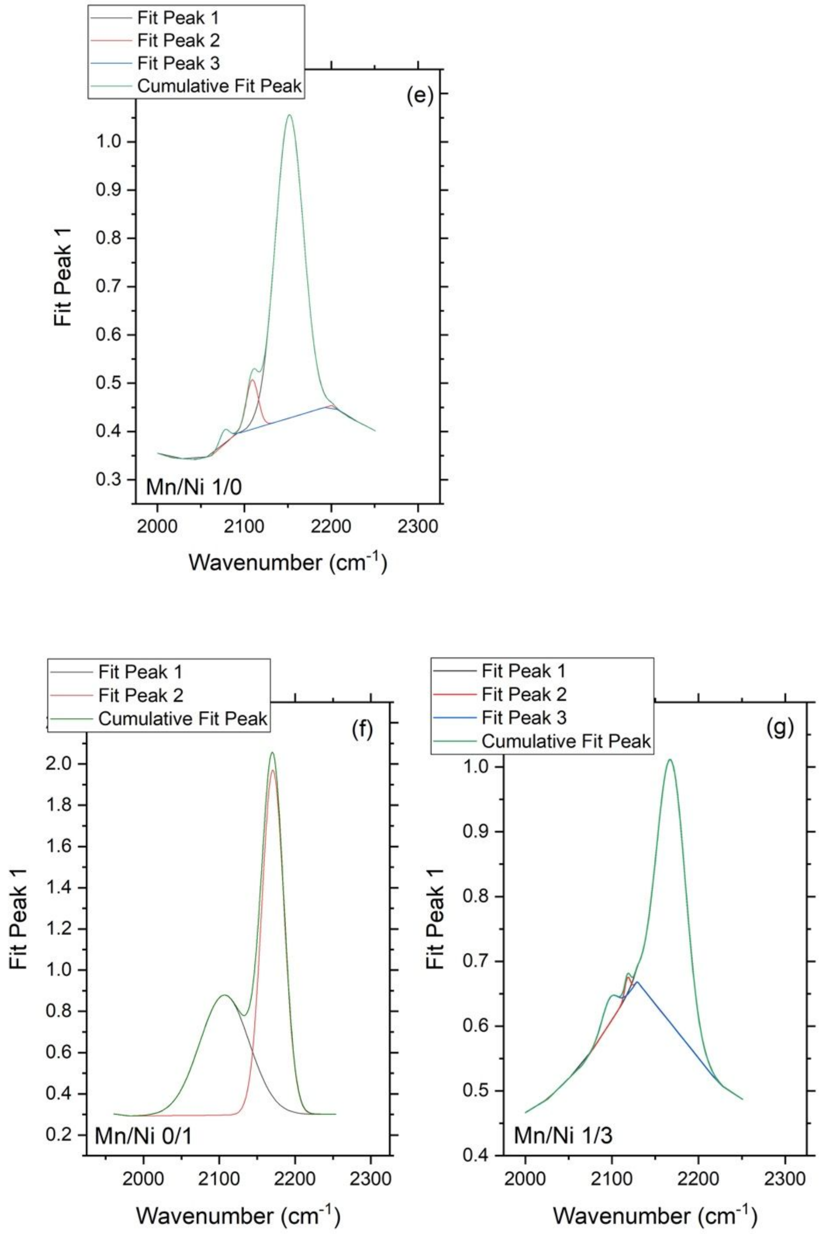
Fit Peak 1
Fit Peak 2
Fit Peak 3
Cumulative Fit Peak
(e)
Fit Peak 1
Mn/Ni 1/0
Wavenumber (cm-1)
Fit Peak 1
Fit Peak 2
Cumulative Fit Peak
(f)
Fit Peak 1
Mn/Ni 0/1
Wavenumber (cm-1)
Fit Peak 1
Fit Peak 2
Fit Peak 3
Cumulative Fit Peak
(g)
Fit Peak 1
Mn/Ni 1/3
Wavenumber (cm-1)

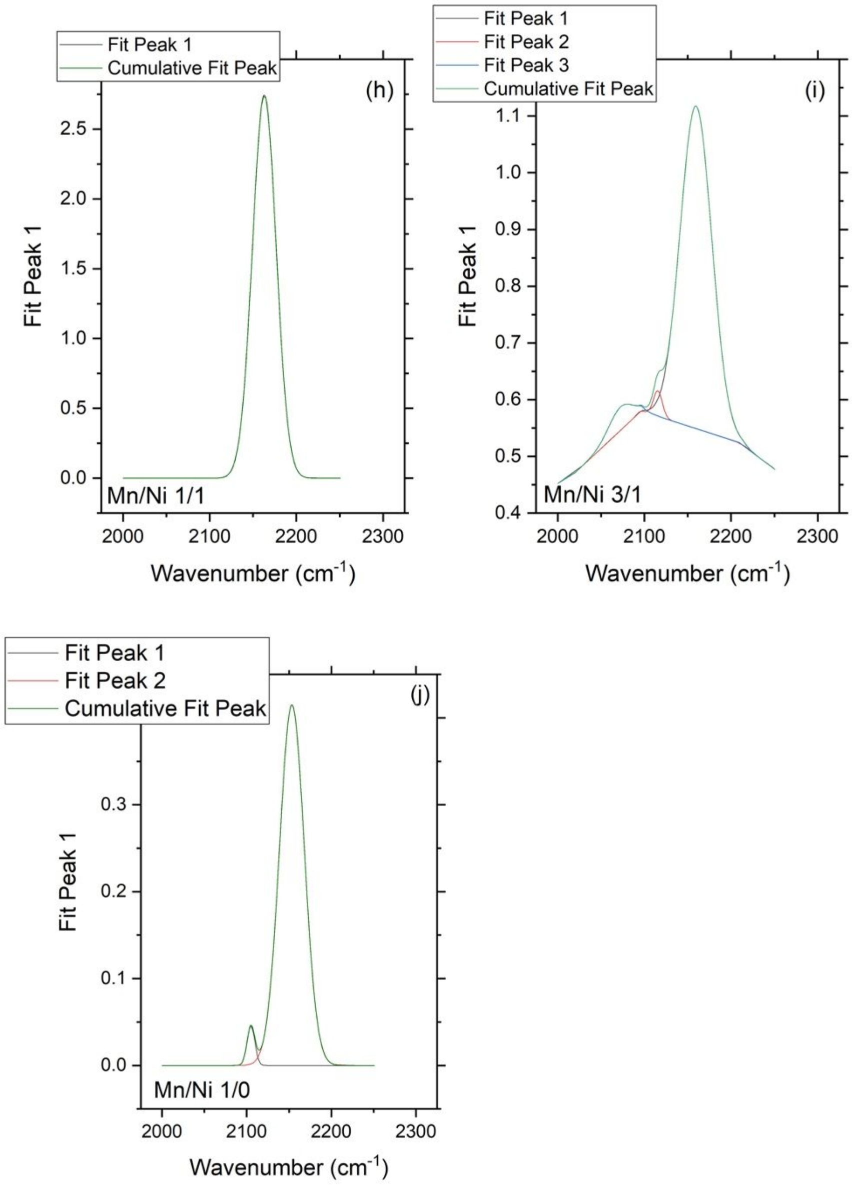

Figure S 4. Peak fitting for the CN stretching of the samples as prepared (a to e) and after 4 h of irradiation with simulated solar light (f to j).

Mn/Ni ratio	Sample label	Fe(III)/Fe(II) ratio	Fe(III)/Fe(II) after sun irradiation
0/1	T3	3.86	8.42
1/3	T8	15.7	4.31
1/1	T6	14.18	∞§
3/1	T7	3.30	10.9
1/0	T5	11.9	29.2
§ *Disappearance of stretching mode Fe(II)-CN.*			

Table S 1. Fe(III)/Fe(II) as calculated from the fitting of FTIR CN vibration mode before and after irradiation.

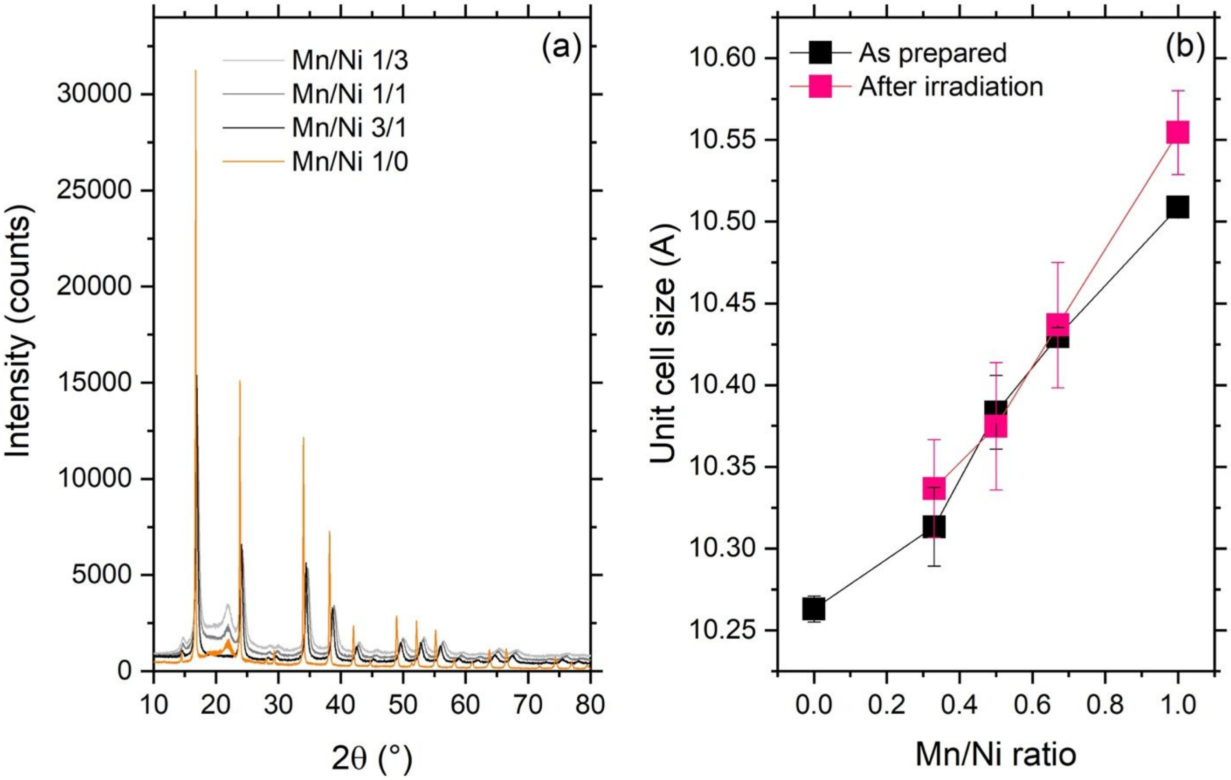

Figure S 5. (a) XRD patterns of the materials after 4h irradiation with simulated solar light and (b) comparison of the unit cell size before and after sun irradiation (markers are experimental points, lines are guide to the eye).

Table S 2. XPS results.

**NiHCF**	**Name**	**C1s - 1**	**C1s - 2**	**Fe2p$_{3/2}$ - 1**	**Fe2p$_{3/2}$ - 2**	**Fe2p$_{3/2}$ - 3**	**N1s - 1**	**N1s - 2**	**Ni2p$_{3/2}$**			**O1s**	**Mn/Ni**
	**Assignment**	**C – C, -CN**	**C – O, C = O**	**[Fe^{III} (CN)6]$^{3-}$**	**Fe – shake-up satellite**	**[Fe^{III}(CN)$_5$H$_2$O]$^{3-}$**	**-CN**	**π-π***	**Ni(+2) in PBA**			**H_2O**	
	Peak BE	285.0	287.4	710.2	714.0	706.5	398.5	401.0	856.7			533.0	
	Atomic %	51.4	3.9	4.9	0.9	0.8	24.4		5.4			6.5	0
**T8**	**Name**	**C1s - 1**	**C1s - 2**	**Fe2p$_{3/2}$ – 1**	**Fe2p$_{3/2}$ – 2**	**Fe2p$_{3/2}$ – 3**	**N1s - 1**	**N1s - 2**	**Ni2p$_{3/2}$**	**Mn2p$_{3/2}$**		**O1s**	
	**Assignment**	**C – C, -CN**	**C – O, C = O**	**[Fe^{III} (CN)6]$^{3-}$**	**Fe – shake-up satellite**	**[Fe^{III}(CN)$_5$H$_2$O]$^{3-}$**	**-CN**	**π-π***	**Ni (+2) in PBA**	**Mn (+2) in PBA**		**H_2O**	
	Peak BE	285.0	287.6	710.1	714.0	706.5	398.4	401.1	856.6	642.3		532.9	
	Atomic %	53.9	5.1	4.2	0.7	0.8	22.9		3.9	0.7		7.6	0.18
**T6**	**Name**	**C1s - 1**	**C1s - 2**	**Fe2p$_{3/2}$ – 1**	**Fe2p$_{3/2}$ – 2**	**Fe2p$_{3/2}$ – 3**	**N1s – 1**	**N1s - 2**	**Ni2p$_{3/2}$**	**Mn2p$_{3/2}$**	**K2p3**	**O1s**	
	**Bond**	**C – C, -CN**	**C – O, C = O**	**Fe (+3) – CN**	**Fe – shake-up satellite**	**[Fe^{III}(CN)$_5$H$_2$O]$^{3-}$**	**-CN**	**π-π***	**Ni (+2) in PBA**	**Mn (+2) in PBA**	**K (+1)**	**H_2O**	
	Peak BE	285.0	287.5	710.2	713.9	706.8	398.4	401	856.4	642.3	293.8	532.8	
	Atomic %	50.7	4.4	4.7	0.5	0.5	26.4		2.9	2.8	0.6	6.4	1.0
**T7**	**Name**	**C1s - 1**	**C1s - 2**	**Fe2p$_{3/2}$ – 1**	**Fe2p$_{3/2}$ – 2**	**Fe2p$_{3/2}$ – 3**	**N1s - 1**	**N1s - 2**	**Ni2p$_{3/2}$**	**Mn2p$_{3/2}$**	**K2p3**	**O1s**	
	**Bond**	**C – C, -CN**	**C – O, C = O**	**Fe (+3) – CN**	**Fe – shake-up satellite**	**Fe (0)**	**-CN**	**π-π***	**Ni (+2) in PBA**	**Mn (+2) in PBA**	**K (+1)**	**H_2O**	
	Peak BE	285.0	287.5	710.2	713.7	706.9	398.4	401.2	856.4	642.0	293.9	532.6	
	Atomic %	47.1	3.9	5.1	0.6	0.4	25.0	1.5	2.4	5.4	0.6	5.3	
**MnHCF**	**Name**	**C1s - 1**	**C1s - 2**	**Fe2p$_{3/2}$ – 1**	**Fe2p$_{3/2}$ – 2**		**N1s - 1**	**N1s - 2**		**Mn2p$_{3/2}$**	**K2p3**	**O1s**	
	**Bond**	**C – C, -CN**	**C – O, C = O**	**Fe (+3) – CN**	**Fe – shake-up satellite**		**-CN**	**π-π***		**Mn (+2) in PBA**	**K (+1)**	**H_2O**	
	Peak BE	285	287.7	710.2	713.1		398.4	400.9		642.3	293.9	532.7	
	Atomic %	54.5	3.2	4.0	0.3		26.1			6.1	0.8	5.0	

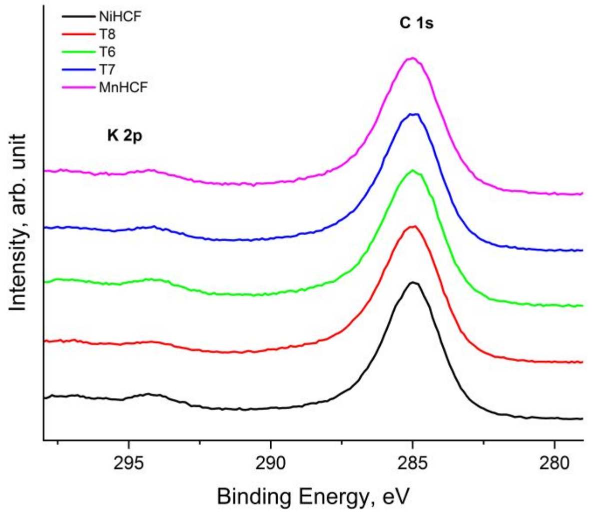

Figure S 6. Comparison of C 1s and K 2p spectra. NiHCF and MnHCF indicate the nickel and manganese binary analogues, respectively.

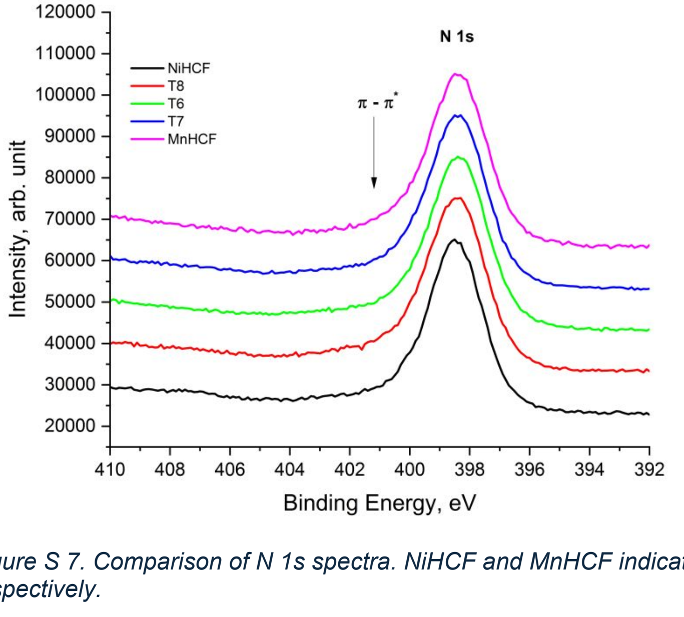

Figure S 7. Comparison of N 1s spectra. NiHCF and MnHCF indicate the nickel and manganese binary analogues, respectively.

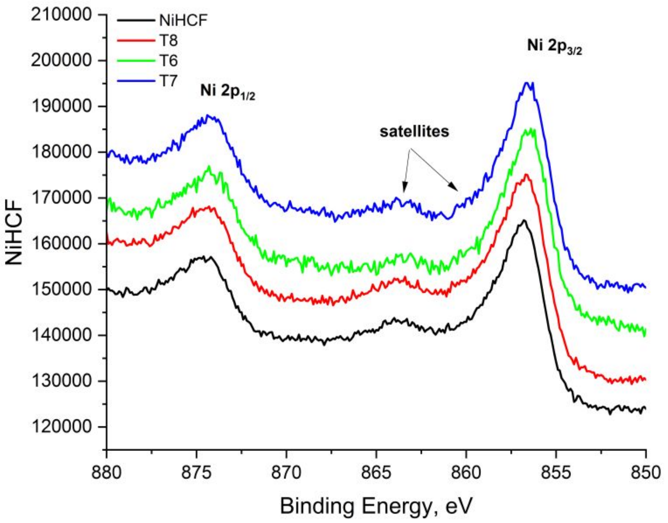

Figure S 8. Comparison of Ni 2p spectra. NiHCF indicates the binary nickel analogue.

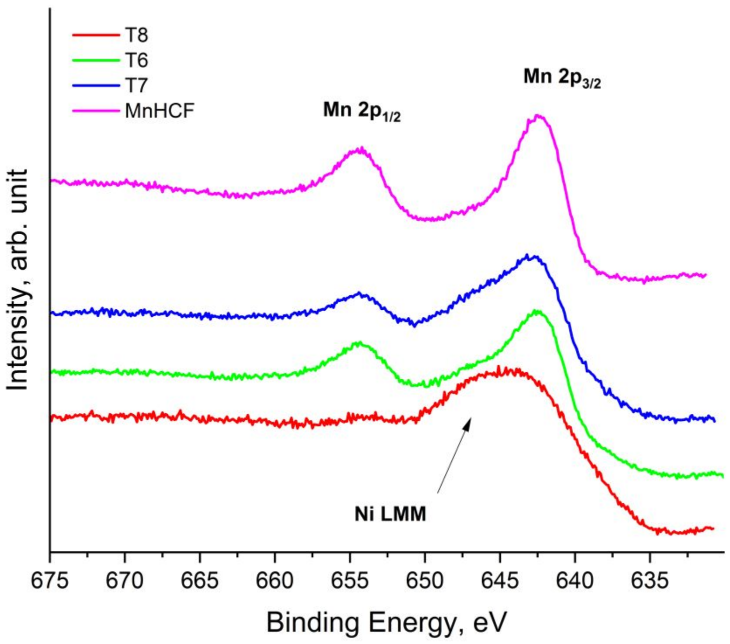

Figure S 9. Comparison of Mn 2p spectra. MnHCF indicates the binary manganese analogue.

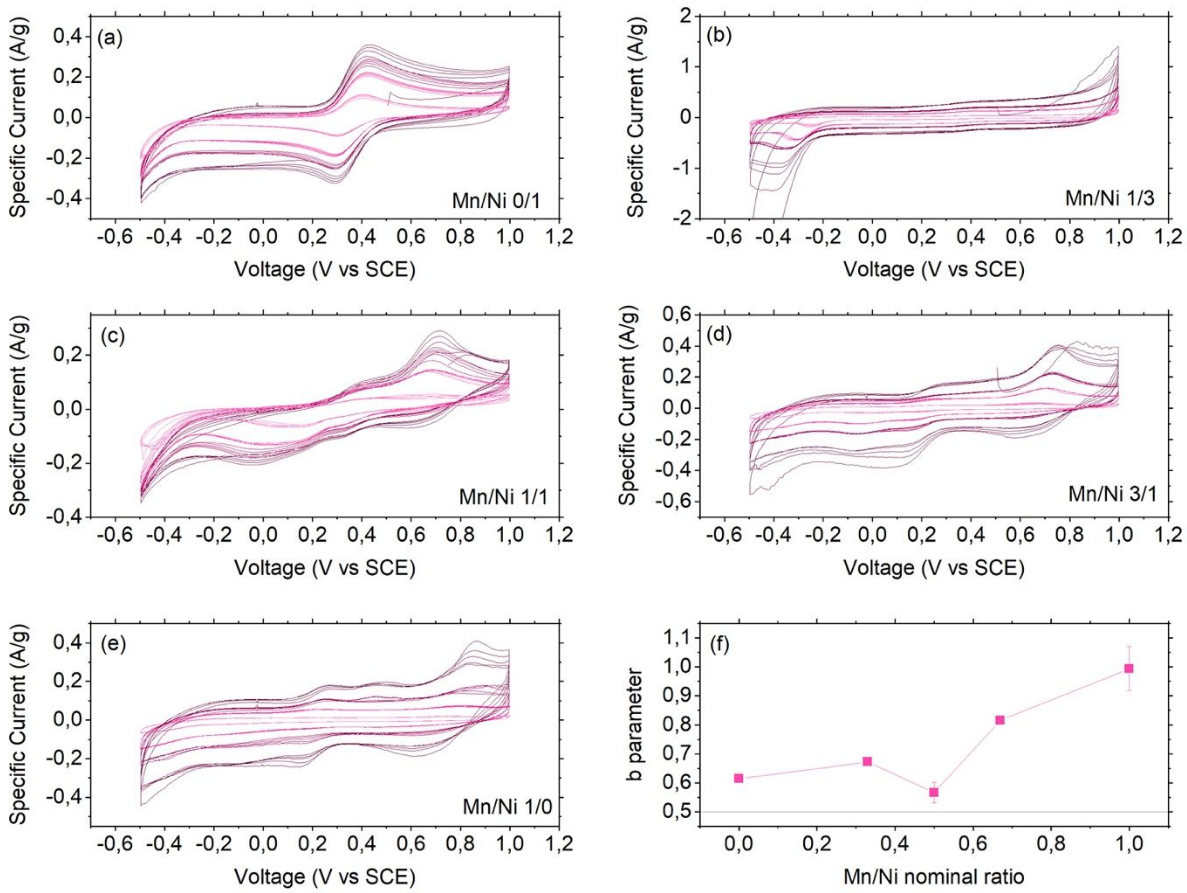

Figure S 10. Cyclic voltammetric curves (a-e) and b parameter (f) in Na_2SO_4 for the materials under investigation.

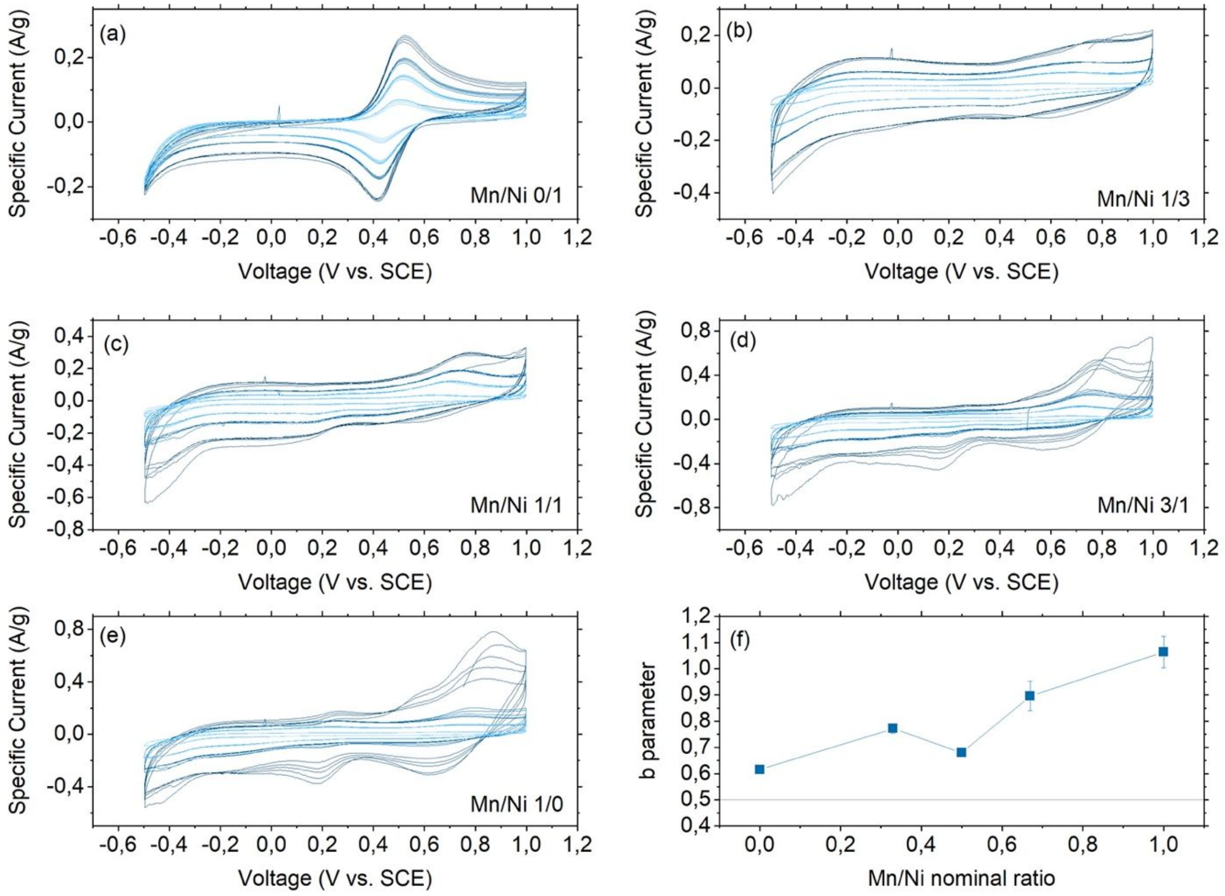

Figure S 11. Cyclic voltammetric curves (a-e) and b parameter (f) in K_2SO_4 for the materials under investigation.

Sample label	Nominal Mn/Ni ratio	OCV Na_2SO_4 (mV)	OCV K_2SO_4 (mV)	Specific capacitance K_2SO_4 (F/g)	Specific capacitance Na_2SO_4 (F/g)
T3	0/1	409.8	524.4	60.3	60.8
T8	1/3	393.6	419.3	42.4	38.7
T6	1/1	379.6	446.6	47.3	46.6
T7	3/1	349.5	418.6	50.9	55.6
T5	1/0	374.4	519.0	52.4	21.6

Table S 3. Electrochemical parameters as retrieved from CV analysis.

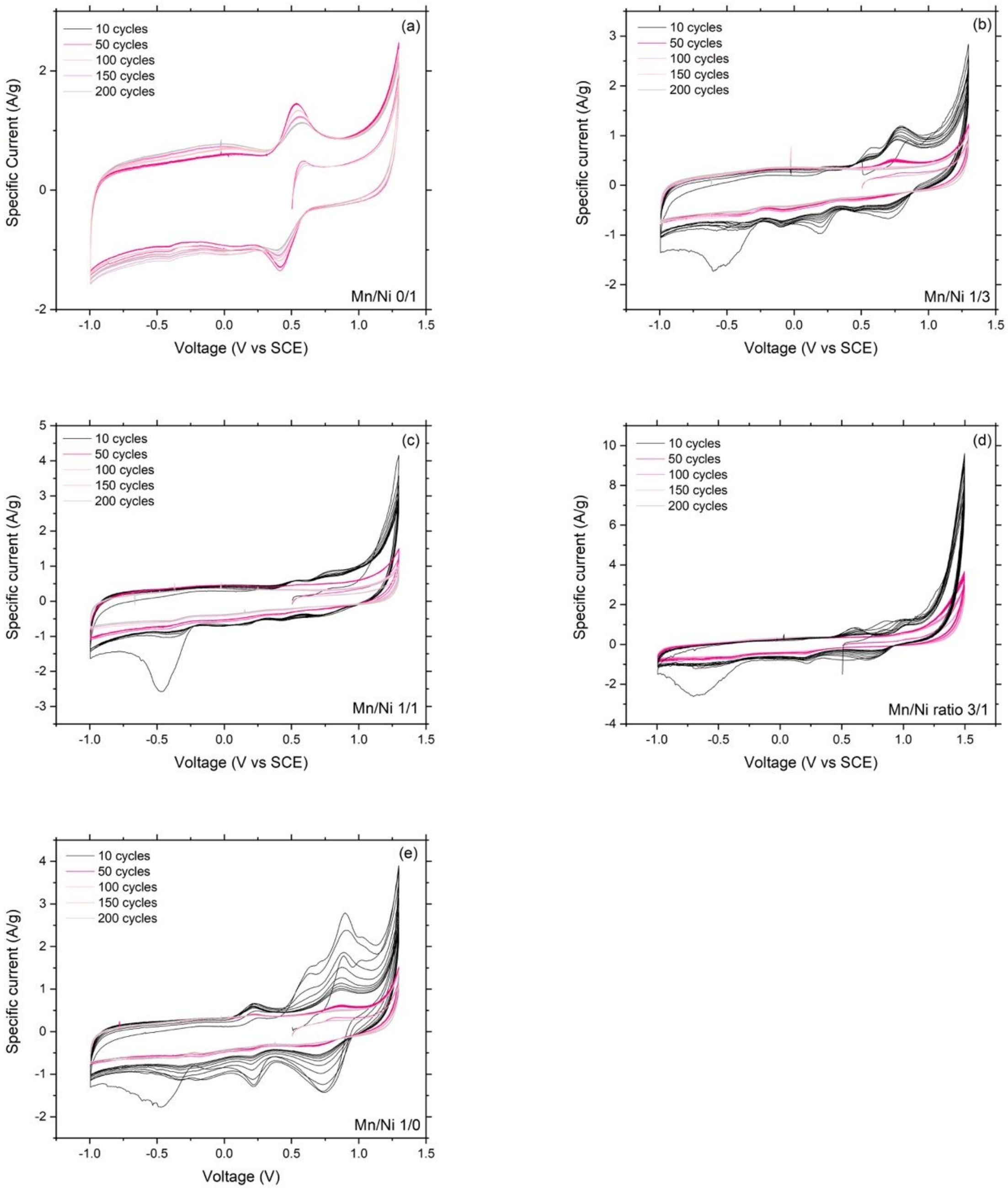

Figure S 12. Long cyclic voltammetric curves (200 cycles), recorded at 100 mV/s in K_2SO_4. (a) to €: increasing amount of manganese content.

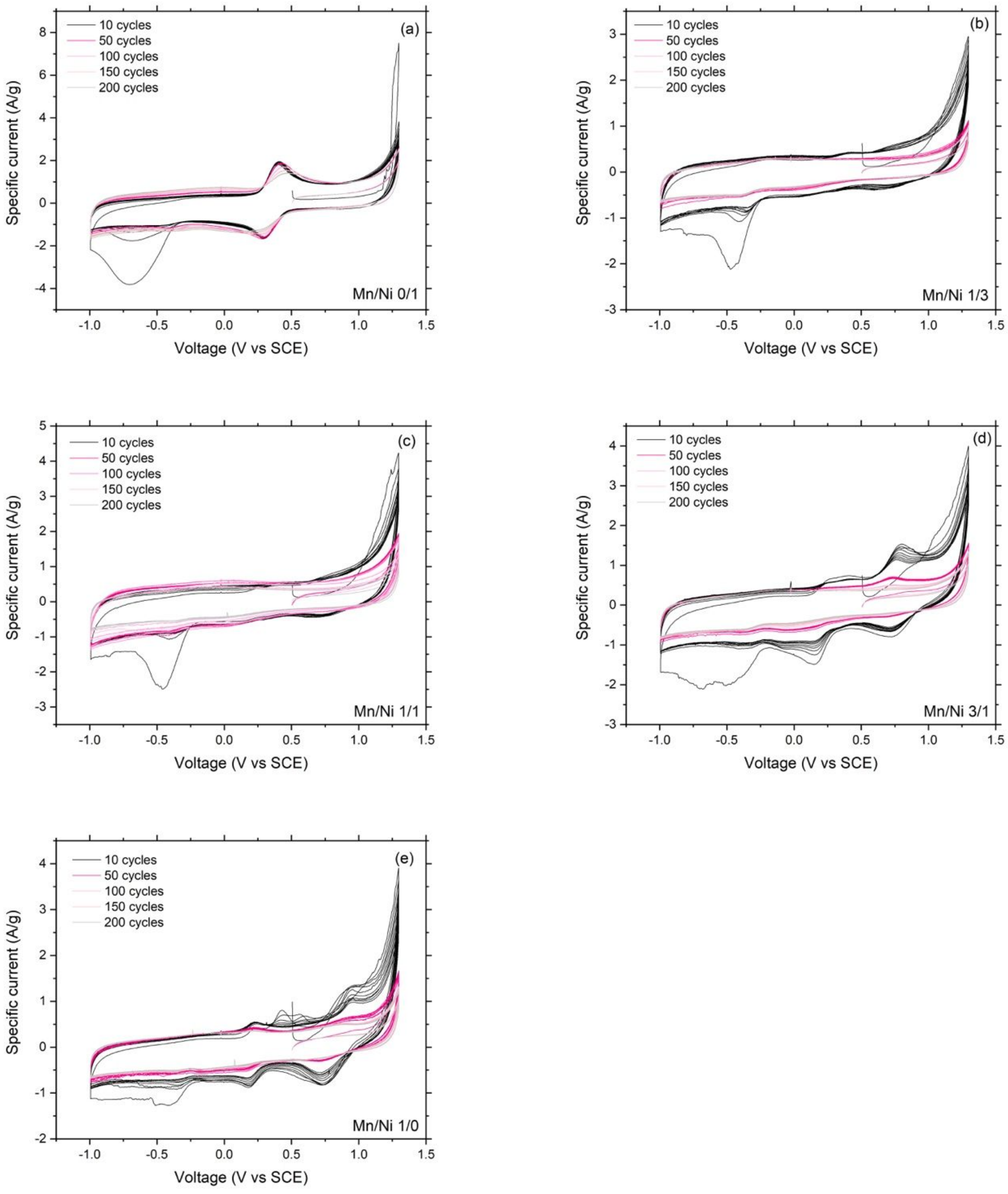

Figure S 13. Long cyclic voltammetric curves (200 cycles), recorded at 100 mV/s in Na_2SO_4

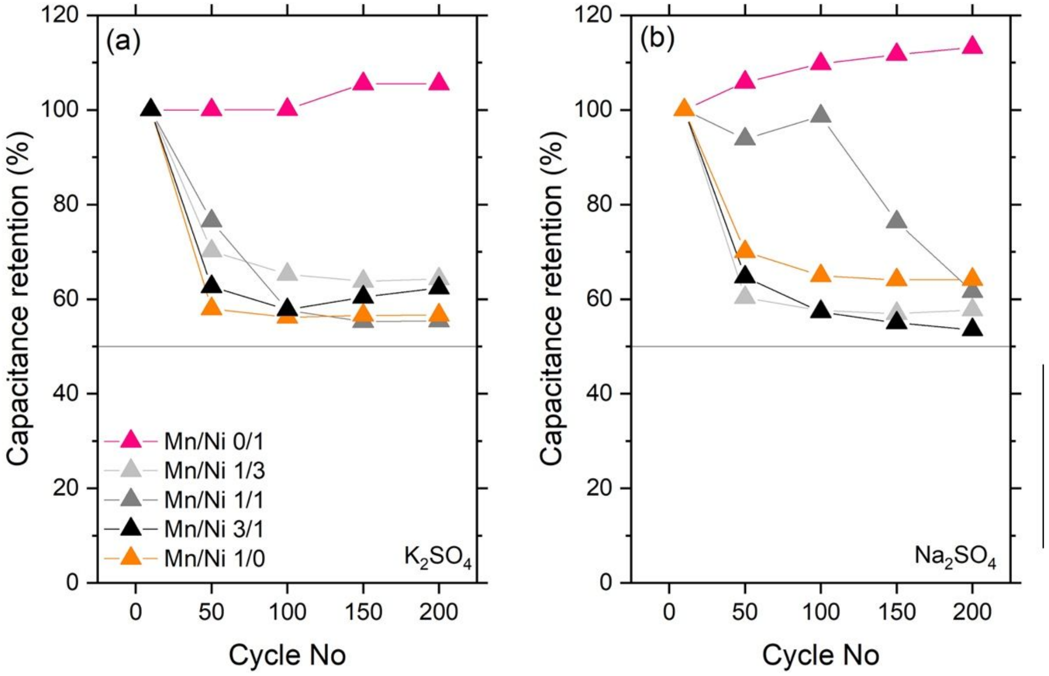

Figure S 14. Capacitance retention in K2SO4 (a) and Na2SO4 (b) over 200 cycles CV at a scan rate of 100 mV/s. A reference line showing a 50% retention is traced for clarity purposes.

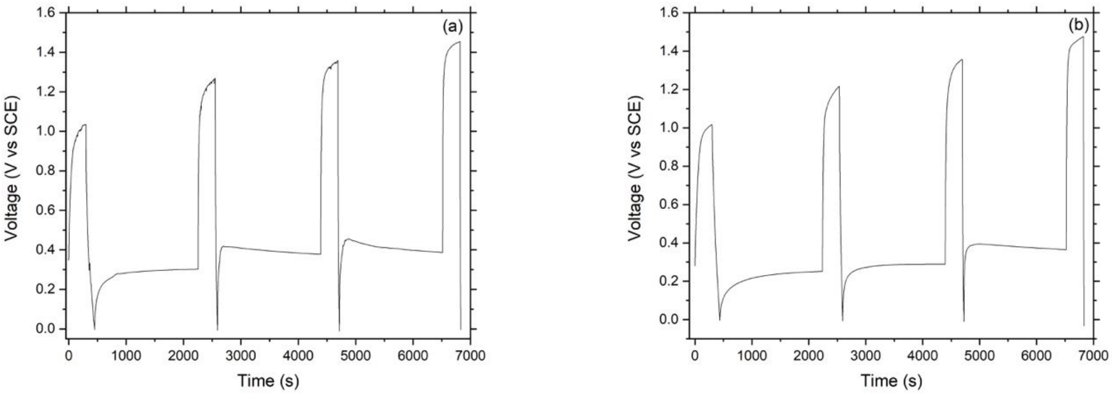

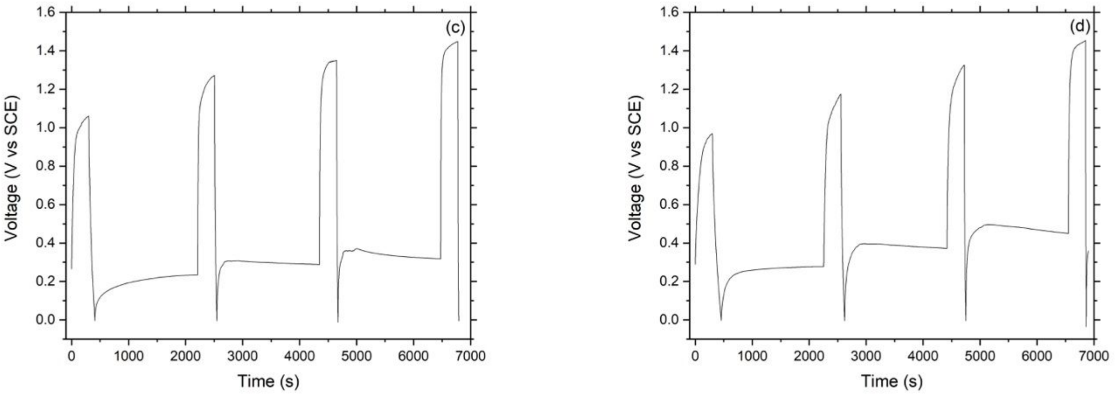

Figure S 15. Galvanostatic charge/discharge curves recorded for the manganese analogues at different constant currents (25.75 mA/g, 77.25 mA/g, 128.75 mA/g, 257.5 mA/g). From (a) to (d): increasing Mn/Ni ratio.

Mn/Ni ratio	Charge – τ (s)	Discharge - τ (s)
1/3	46.30 ± 0.4778	63.42 ± 2.713
1/1	50.30 ± 0.2059	118.4 ± 2.134
3/1	38.44 ± 0.3462	69.09 ± 1.512
1/0	71.63 ± 0.1960	105.0 ± 2.168

Table S 4. Galvanostatic charge/discharge time constants, retrieved by exponential fitting of the GCD curves recorded at I = 25.75 mA/g.
